# Oligothiophene-*g*-oligo(D,L-lactide) “Rod-*Graft*-Coil” Co-Oligomer: Self-Assembly Modulation by Processing and Formed Films Surface Morphological Diversity Characterization †

**DOI:** 10.3390/polym18212724

**Published:** 2026-11-02

**Authors:** Anca-Dana Bendrea, Demet Göen Colak, Luminita Cianga, Mariana Pinteala, Ioan Cianga

**Affiliations:** 1 Centre of Advanced Research in Bionanoconjugates and Biopolymers, ”Petru Poni” Institute of Macromolecular Chemistry 41 A, Grigore-GhicaVoda Alley, 700487 Iasi, Romania; anca.bendrea@icmpp.ro (A.-D.B.); pinteala@icmpp.ro (M.P.); 2 Department of Chemistry, Faculty of Science and Letters, Istanbul Technical University, Maslak, 34469 Istanbul, Turkey; goende@itu.edu.tr

**Keywords:** conjugated polymers, graft copolymers, polymacromonomers, self-assembling, oligothiophenes, oligo(D,L-lactide), thin films, morphology control

## Abstract

In the field of advanced functional materials, π-conjugated, electrically conductive polymers (CPs) represent a highly specialized class, intensively studied as essential in contemporary optoelectronic and biointerfaced technologies. The solution-processability of CPs represents a significant advantage; however, the interactions occurring in this state make it difficult to establish suitable strategies for controlling the resulting solid-state microstructures and the charge transport. In this context, the main aim of this paper was to analyze how the polymer architecture and other deliberately chosen design criteria, can be harnessed and easily combined with a versatile processing toward self-assembled thin films of varying surface properties. Thus, a “rod-*graft*-coil” polymacromonomer, denoted OTh-PDLLA,—previously synthesized via metal-free-photoinduced oxidative polymerization, yielding an oligomeric structure comprising a π-conjugated thiophene backbone with densely grafted oligo(D,L-lactide) side chains—was processed into thin films by drop-casting, in native and also in its LiClO_4_-treated form, on rigid glass slides and on flexible polylactide (PLA) sheets, using solvents of differing quality, at various concentrations. The influence of these processing parameters on the shape and size of the formed self-assembled supramolecular structures on films surface was analyzed, and the processing-dependent surfaces wettability changes were also discussed.

## 1. Introduction

The importance of π-conjugated polymers (CPs), well known as electrically conductive polymers, has evolved since their introduction to the field of polymer and materials science (in the mid-to-late 1970s) [1], shifting from a subject of challenging academic interest to one that encompasses a global market valued at USD 3.6 billion in 2023 and projected to reach USD 7.1 billion by 2030 [2]. This evolution is closely correlated with the increasing dependence of human society on consumer electronics, concomitantly with increasing integration of CPs into almost all modern optoelectronic devices due to their critical role in enabling device miniaturization, and energy efficiency, besides many other benefits [3]. Currently, it is highly desirable for these CP-based materials to be seamlessly integrated with the environment and interfacing with living systems without any adverse impact as there is a surge of interest on wearable [4] and implantable electronics [5]. Even if CPs become technologically mature in the field of optoelectronics [2,3], they have also successfully entered the field of synthetic polymers-based biomedical materials. Thus, they performed as sensing, imaging and therapeutic strategies for precision medicine [6] and also as scaffolds or coatings for tissue engineering [7,8]. Importantly, as CPs are the materials of choice in the field of bioelectronics [9], the possibility to fabricate living electronic interfaces fully integrated into animal tissues [10] or a bio-hybrid photosynthetic living system [11] are examples of their emerging applications. The macromolecular design, besides the understanding of processing parameters, is the key element to adapt new CPs to next-generation technologies that prioritize biocompatibility, sustainability, and adaptability [12]. To take the advantage of macromolecular architecture for functions tailoring can be an inspired option as segmented rod-coil CPs copolymers proved [13]. Due to the presence of rigid segments, they exhibit a distinct self-assembly in solution into nanoscale objects with various morphologies [14], offering custom nanomaterials for diverse fields of application [13].

As a result of many complex interactions, especially in the presence of solvents [15], CPs are characterized by a hierarchical multi-level self-assembly, at dimensional scale, evidencing quaternary structures as the protein does [3]. In fact, as early as several decades ago, the supramolecular self-assembly behavior of rod-coil molecules (including π-conjugated rods) and the formed nanostructures having specific shape, mainly influenced by the rigidity contrast between the two molecular components, was already stated as an interesting analogy with folded proteins [16,17].

This analogy was extended further by considering that, just as the quaternary structure of proteins contributes to determining their highly specific biological functions [18], the self-assembled supramolecular morphology of conjugated polymers (CPs) directly influences the optoelectronic properties and performance of the resulting devices [19].

The challenges of guiding this multi-level self-assembling of CPs from the solution to the thin films, and of the accurate understanding of the structure–property relationships, grow as the complexity of CPs’ structure does. This is the reason why this type of study regarding rod-coil grafted CPs does not appear so often in the literature. In the available reports it was pointed out that, besides the architectural effect on the rod-*g*-coil CPs’ solution self-assembling [20,21], the shape and size of the formed supramolecular structures essentially depend on the processing parameters (like solvent quality, concentration and the support nature [20], pH [22,23], temperature [23]).

**Scheme 1:**
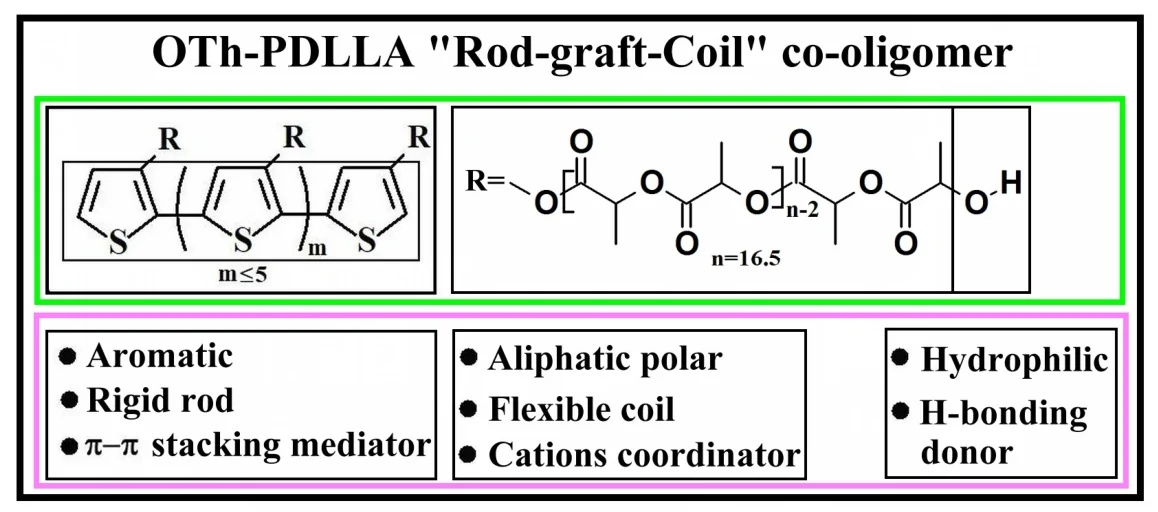
Molecular structure of OThPDLLA cooligomer and its features relevant for self-assembling.

In one of our previous papers we reported the synthesis and characterization of a polymacromonomer denoted OTh-PDLLA [24] (Scheme 1), starting from its homologous macromonomer, by a metal-free-photoinduced oxidative polymerization. After polymerization, a rod-*g*-coil co-oligomer resulted, with a π-conjugated oligothiophene main chain densely grafted with oligo(D,L-lactide) side chains. OTh-PDLLA was intentionally designed to optimally work in aqueous electrolytes as body fluids-mimicking media [25] and for this aim, to the electron conducting thiophenic main chain the polar side chains PDLLA were deliberately attached as they contain ester moieties with the capability to coordinate ions [26], creating favorable conditions for their movement for a better interaction with ions-containing living systems.

Moreover, these side chains show multifunctionality due to their well-known biocompatibility and sensitivity to hydrolytic degradation, endowing the whole structure of co-oligomer with partial degradability.

Because surface properties also directly impact on the biological events developed at the film’s biointerface, it was considered important to follow OTh-PDLLA co-oligomer capability for forming thin films, on either rigid or flexible supports compatible with biological milieu and to investigate how the properties of the new material could be handled to obtain the optimum performance for a certain application. Thus, from the important environmental variables of processing, the type of solvent and the concentration were chosen because these two parameters impact the conformation of both the side chains and the conjugated main chain in solution with direct implications on the morphology and surface properties of formed films. In addition, the thin films self-assembling behavior of OTh-PDLLA was followed on the support of diverse rigidity, this being an important aspect if the applications as functional surface coatings are envisaged.

For films deposition, the drop-casting technique was preferred in spite of the obtained film’s thickness heterogeneities. This method’s benefit, when applied into a solvent saturated atmosphere (as detailed in the experimental section), is that it allows for slow, solvent evaporation, concomitantly enabling the polymer self-assembling without any other external force intervention.

From the organic solvents, chloroform (Chl) and dimethyl sulfoxide (DMSO) were selected, considering their Hansen solubility parameters (δ) and Flory–Huggins polymer-solvent interaction parameters (χ), (Table S1), relative to those of the PDLLA side chains only, since oligothiophenes are well known to be insoluble in any organic solvent. As the values of solubility parameters (δ) and the solvent–polymer interaction parameter (χ) in Table S1 show, the two solvents chosen for the investigations are essentially different. Thus, Chl, which behaves as a non-polar solvent, is commonly used for PLA processing in different form factors, (films, membranes, fibers by electrospinning, etc.) [27]. In contrast, polar DMSO having a high boiling point and a higher value of solvent–polymer interaction parameters (χ) (Table S1) was used being considered of greener character, in addition to the reported pharmaceutical nature and therapeutic properties [28]. DMSO works as a weaker solvent than Chl for the side chains attached to the oligothiophenic backbone. However, in the particular case of OTh-PDLLA, due to its oligomeric nature, both in terms of the side chains and the backbone, and also of the low values of the concentrations used for films casting, no dissolution deficiencies were noticed in DMSO; while the obtained dispersions kept their transparency within several months in laboratory conditions without formation of turbidity and precipitation. These experimental observations are in line with the previously reported cases when grafted copolymers with PLA side chains with the comparable polymerization degree were easily dissolved in DMSO at comparable or even higher concentrations [29].

On the other hand, we took the advantage of DMSO solvent quality sensitivity on the molecular weight of PLA [30] and used it for OTh-PDLLA deposition (in native and LiClO_4_-treated forms) onto the flexible, biocompatible and biodegradable sheets of high molecular weight PLA. Even though the support surface may swell slightly during solvent evaporation and film formation during the processing and film casting on this support, no such phenomenon was noticeable by the naked eye at the macroscopic level.

The logic behind this study was to observe how more extended (in Chl) or more collapsed (in DMSO) PDLLA side chains influence the conformation and shape of the thiophenic conjugated main chain when dispersed into solvents and ultimately how the variation in the considered parameters is translated into the morphology and surface properties of the obtained films on different supports. Another processing parameter influencing self-assembling that was considered in our experiments was the treatment of OTh-PDLLA in bulk by solution mixing with LiClO_4_ as a post-polymerization physical modification. The role of the PDLLA side chains in this process was described in a detailed manner.

## 2. Experimental Section

### 2.1. Materials Used for Synthesis of OTh-PDLLA Polymacromonomer and for PLA Support

3-thiophene-methanol (Th-Me), 3,6-dimethyl-1,4-dioxane-2,5-dione (or rac-lactide, the 50:50 racemic mixture of D- and L-Lactide, accordingly with provider description), tin(II)-2-ethylhexanoate (Sn(Oct)2) and diphenyliodonium hexafluorophosphate (DPI) (>98%), lithium perchlorate (LiClO_4_), (all from Merck-Sigma-Aldrich, Darmstadt, Germany) were used as received. PLA IngeoTM Biopolymer 2003D (NatureWorks LLC, Minnetonka, MN, USA) was obtained from renewable resources, with a 4% content of D-lactide and was supplied by Nature Works LLC (Minnetonka, MN, USA), in the form of pellets, having a specific gravity of 1.25 g cm^−3^, M_w_ of 1.43 × 10^5^ g mol^−1^, M_n_ of 7.54 × 10^4^ g mol^−1^, and a dispersity index (Ð_M_) of 1.88; it was used for PLA sheets obtainment.

### 2.2. Synthesis of OTh-PDLLA Co-Oligomer

By applying the “grafting through” technique, the OTh-PDLLA polymacromonomer investigated in the present paper was synthesized via *metal-free* photoinduced oxidative homopolymerization and the experimental details besides detailed structural characterization were previously reported [24]. The obtained grafted co-oligomer structure was proved by ^1^H-NMR and FTIR spectroscopy techniques. The characteristic signals for both the thiophenic main chain and oligolatide side chains were found in the corresponding spectra and the main ones are as follows: ^1^H-NMR (400 MHz, CDCl3), δ (ppm): 7.71 (1H, multiplet, chain-end thiophene ring); 7.53 (1H, multiplet, chain-end thiophene ring); 7.05 (m + 2 H, multiplet, 3-substituted-thiophene-2,5-diyl structural units); 5.0–5.5, 4.2–4.5 and 1–2 ppm for protons of PDLLA side chains (see reference [24] for detailed spectrum interpretation). IR (KBr), cm^−1^: 3112 (ν(C-H)_α_ + ν(C-H)_β_ thiophene rings at the main chain ends); 2998 (ν_as_CH_3_); 2943 (ν_s_CH_3_); 1756 (νC=O); 1188 (ν_as_ C-O-C); 1090 (ν_s_ C-O-C); 892, 862, 831, and 699 (thiophene rings); 1621 (typical for the conjugation between thiophene rings along the oligomeric chain). The number average molecular weight of OTh-PDLLA obtained by GPC measurement was M_GPC_ = 4508, while the polydispersity index IPD = 2 is typical for a step-growth polymerization. It is also important to highlight that, by calculation from the ^1^H-NMR data, the OTh-PDLLA co-oligomer’s polymerization degree (PD) of 6.9 was established, while by specific investigation it was approximated that most of the chains are pentaoligomers [24].

### 2.3. In Bulk Modification of OTh-PDLLA with LiClO_4_ by Solutions Mixing

The post-processing of OTh-PDLLA co-oligomer with LiClO_4_ was performed by mixing a dispersion of OTh-PDLLA in chloroform (Chl) with a solution of LiClO_4_ dissolved in THF. As an equimolar ratio between co-oligomer and salt was intended, the molar concentration of OTh-PDLLA was calculated based on the molar mass of its repeating unit (RU). As such, the value of number average molecular weight obtained by calculation from ^1^H-NMR data of the Th-PDLLA macromonomer was considered (M_n-H-NMR_ = 2489) [24]. For solution mixing, at 1 mL of OTh-PDLLA (1 mg/mL dispersion in Chl), 1 mL of LiClO_4_ in THF, at concentration of 0.043 mg/mL, was added. The obtained mixture was magnetically stirred at room temperature for 24 h, being subsequently left in the fume hood to evaporate all the organic solvents. The solvents’ traces that eventually remained after evaporation were further removed in a vacuum oven for another 24 h at room temperature. The resulted modified oligomer denoted as D-OTh-PDLLA was washed with Milli-Q water several times to ensure that the excess of uncoordinated or non-intercalated salt has been removed. Finally, the resulted powder was dried in a vacuum oven overnight, also at the room temperature and then the mass of the resulting salt-treated D-OTh-PDLLA was determined by weighing. The calculated salt loading by OTh-PDLLA was determined as 91%, expressed as the percent from the initial LiClO_4_ feed amount.

### 2.4. OTh-PDLLA Thin Films Preparation

#### 2.4.1. Preparation of Substrates Used for OTh-PDLLA Film Deposition

For OTh-PDLLA thin films deposition two types of supports were used. Thus, the round glass slides obtained from Paul Marienfeld GmbH@Co.KG, Lauda-Königshofen, Germany, having a diameter of 15 mm, were used after a prior cleaning in an ultrasonic bath in cycles of 5 min successively with distilled water, ethyl alcohol, and acetone. Subsequently the round glass slides were dried in vacuum.

The PLA sheets with a thickness between 0.25 and 0.3 mm were obtained in accordance with a protocol previously described [31]. Thus, the neat PLA pellets were hot-pressed with a LabTech LP-20B hydraulic press (LabTech, Samut Prakan, Thailand) at 175 °C, applying a preheating time of 3 min and a pressing time of 3 min at 140 bars followed by a sudden cooling of the mold under pressure.

#### 2.4.2. Self-Assembly of OTh-PDLLA in Organic Solvents and Thin Films Obtainment

Co-oligomer in a native state (OTh-PDLLA) or in its lithium perchlorate (LiClO_4_)-treated form, (D-OTh-PDLLA), was self-assembled in Chl or in dimethyl sulfoxide (DMSO) by simple direct dissolution method [20]. Thus, OTh-PDLLA was dispersed in the mentioned solvents at final concentrations of 1 mg/mL and 10 mg/mL, and the obtained samples were denoted as OTh-PDLLA-1 and OTh-PDLLA-10, respectively. D-OTh-PDLLA was dispersed in the same solvents, but at a concentration of only 1 mg/mL. In order to assess the influence of processing parameters on the formed films’ surface morphology, subsequently 30 µL of these dispersions were deposited onto the substrates of similar area, by using the drop casting technique, as follows: (i)—on the glass slides both OTh-PDLLA-1 and OTh-PDLLA-10 from both solvents; (ii)—on PLA sheets OThPDLLA-1 and OTh-PDLLA-10 from only DMSO; (iii)—D-OTh-PDLLA on glass from both solvents and (iv)—D-OTh-PDLLA on PLA sheets from only DMSO. Regardless of the used support, to assure the uniform solvent evaporation, the samples were kept in a solvent-saturated atmosphere during film formation by using a glass dome that only partially covered them and dried at ambient temperature. Prior to all investigations, the resulted films were vacuum-dried for 24 h to remove the solvent traces and/or eventually any moisture. The obtained films are thickness-heterogeneous ones as can be seen in Figure S1.

### 2.5. Measurements

Particles size measurement was carried out by dynamic light scattering (DLS) using a Malvern Zetasizer Nano ZS instrument (Malvern Instruments Ltd., Malvern, UK) equipped with a 4.0 mW He–Ne laser operating at 633 nm and a detection angle of 173°. Samples were used as prepared, without filtration at two different concentrations, c = 10 mg/mL and c = 1 mg/mL, respectively, in different solvents. The reported values represent the average of three measurements made for each sample, at 25 °C with an equilibration time of 5 min before starting each measurement. Quartz cells were used for organic solvents. Zetasizer7.13 Software was used for data processing. The measurements were performed in triplicate for each sample and the results are presented as average ± S.D.

Atomic Force Microscopy, (AFM), was used to visualize all the OTh PDLLA and D-OTh-PDLLA obtained films morphology using a NTEGRA Scanning Probe Microscope (NT-MDT Spectrum Instruments, Zelenograd, Moscow, Russia) with NSG10 high-resolution AFM cantilever (TipsNano OÜ, Tallinn, Estonia). The free resonant frequency of the cantilever was 169 kHz, and the corresponding cantilever’s normal spring constant was 6.98 N/m. The apparatus was operated in semi-contact mode, over scanning areas relevant to the surface morphology corresponding to each of the studied cases. The AFM data acquisition was performed using Nova 1.1.1.19891, each row image being flatten corrected using 2nd polynomial order fit lines. The 3D texture parameters (Sq—root mean square roughness, Sdr—developed interfacial area ratio) were calculated based on the obtained processed images using Image Analysis 1.0.0.0. Both aforementioned softwares were developed by NT-DMT Spectrum Instruments, Zelenograd, Moscow, Russia. The mean and standard deviation values for the Sq, Sdr, and roughness parameter (r) derived from Sdr, were computed based on 3 different scans on the same sample, using Microsoft Excel program.

The wettability of all the surfaces was investigated by measuring the static water contact angle (WCA) using a DataPhysics OCA 15 (Data Physics GmbH, Filderstadt, Germany) optical contact angle goniometer. The contact angle was determined under ambient conditions within 5 s after placing 1 µL drops of deionized water on the sample’s surface. The images of the water droplets were recorded and analyzed using the software provided by the manufacturer to determine the contact angle values. At least three measurements (six contact angle values) were performed at randomly selected locations across the sample’s surface.

FTIR spectra were recorded on a Bruker Vertex 70 FTIR spectrometer (Bruker Optics GmbH, Ettlingen, Germany) equipped with a diamond ATR device (Bruker OPTIK GmbH, Ettlingen, Germany) in transmission mode. ATR accessory was used for the spectroscopic analysis of the thin films in a wave number range from 4000 to 400 cm^−1^. For a spectrum 64 scans were taken, with a baseline correction. The films used for the study of the LiClO_4_-treatment effect were prepared from Chl, at a concentration of 1 mg/mL and drop cast on a glass slide. UV–vis spectra of OTh-PDLLA and D-OTh-PDLLA were carried out by using a Specord 200 Analytik Jena spectrophotometer (Analytik Jena AG, Jena, Germany) in Chl at c = 1 mg/mL.

## 3. Results and Discussion

### 3.1. OTh-PDLLA’s Self-Assembly Modulation by Processing and Formed Films Surface Characterization

**Figure 1:**
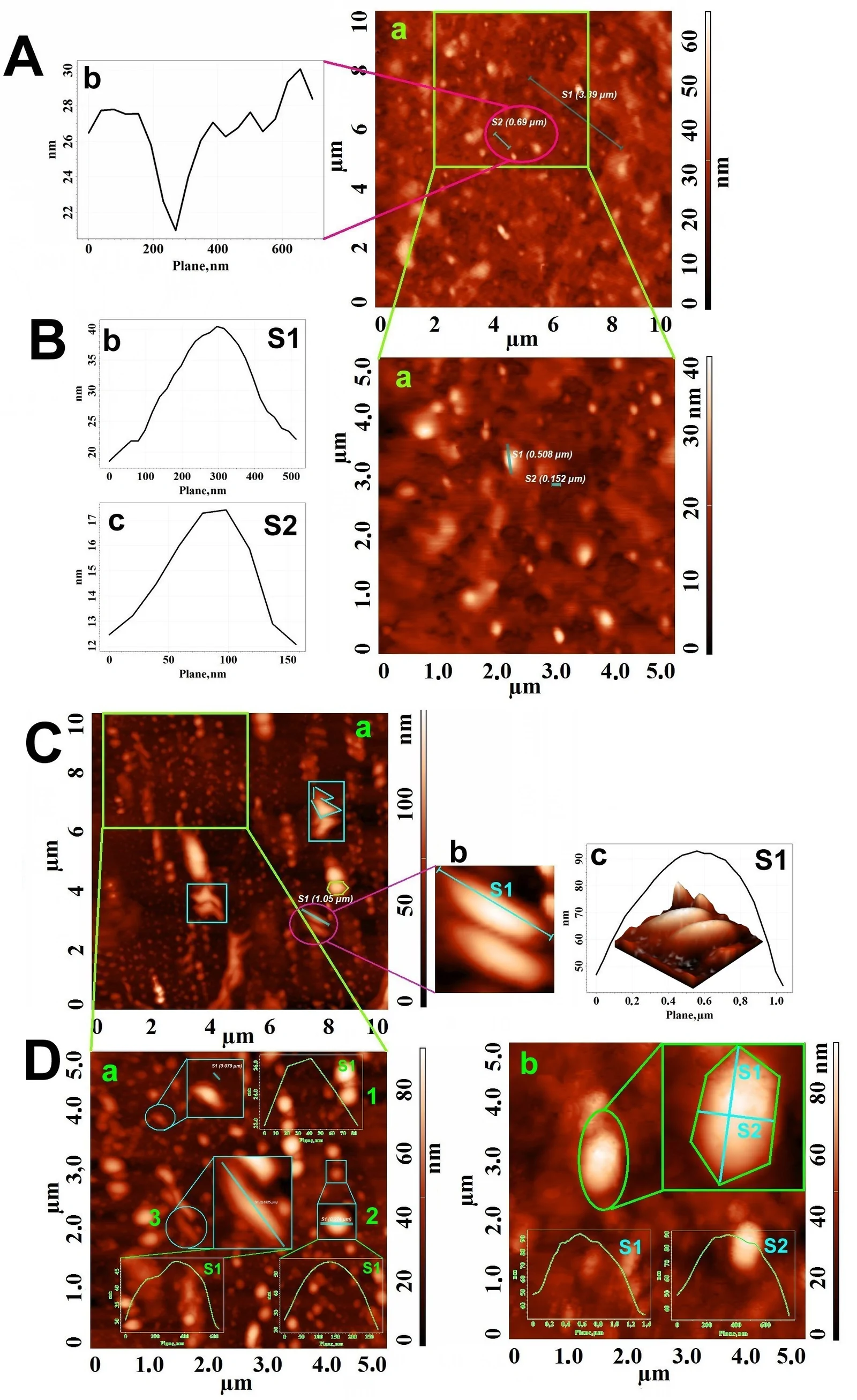
AFM height images and cross-sectional analysis of nanostructures formed on film surfaces obtained by casting OTh-PDLLA-1 from Chl (**A**,**B**) and DMSO (**C**,**D**), on glass slides.

**Figure 2:**
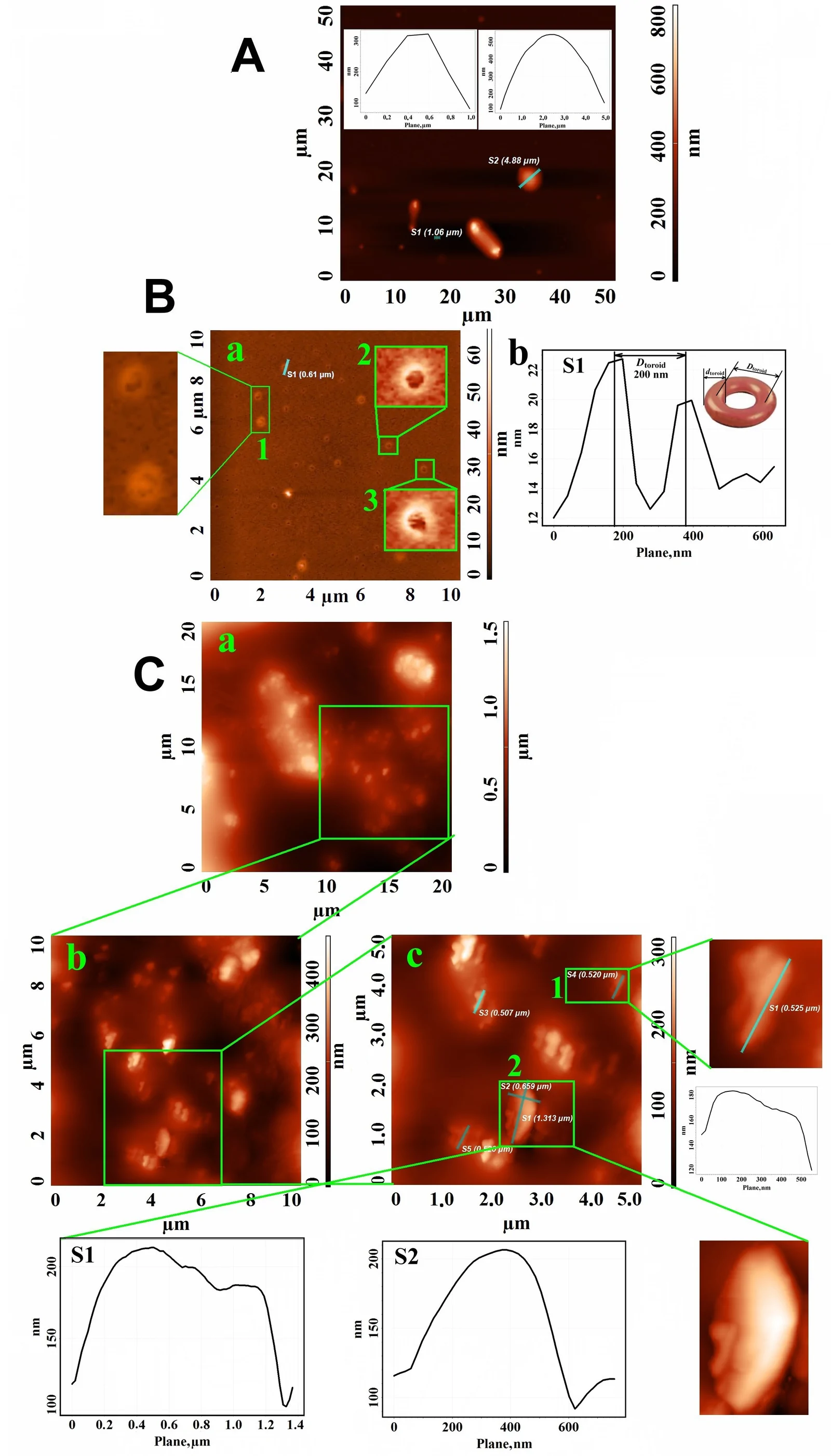
AFM height images and cross-sectional analysis of nanostructures formed on film surfaces obtained by OTh-PDLLA-10 cast from Chl (**A**,**B**) and DMSO (**C**), on glass slides.

**Figure 3:**
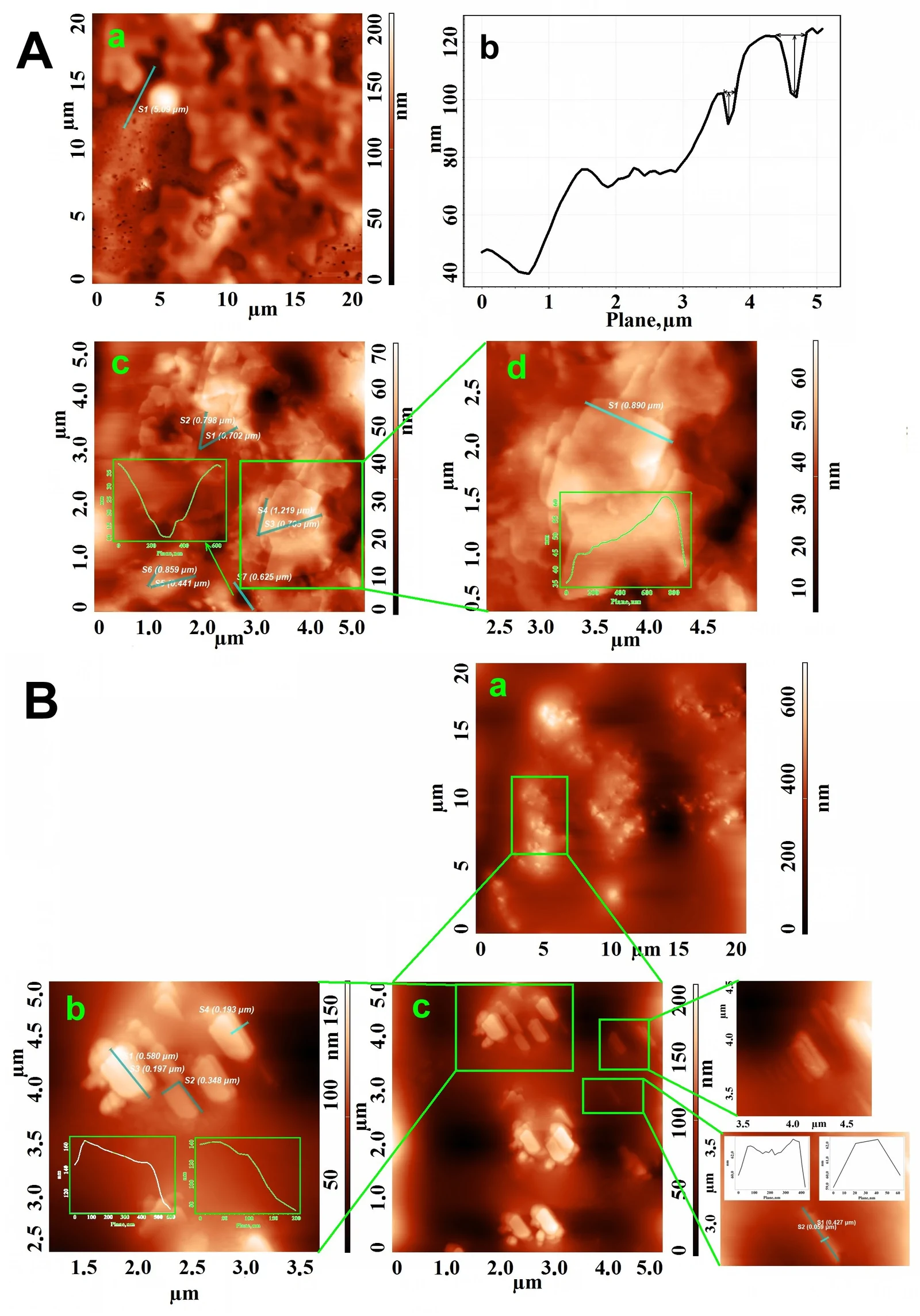
AFM height images and cross-sectional analysis of nanostructures formed on PLA sheets by casting OTh-PDLLA-1 (**A**) and OTh-PDLLA-10 (**B**) from DMSO.

This part of the paper presents the results referring to the films of native OTh-PDLLA, obtained by using both Chl and DMSO as solvents, at values of 1 and 10 mg/mL for concentration when casting on round glass slides, while on PLA sheets the films were obtained from only DMSO at both concentrations. The noticed peculiarities and differences induced by the type of solvent, concentrations and type of supports on the obtained films were evaluated by dynamic laser scattering (DLS), atomic force microscopy (AFM) and water contact angle (WCA). The experimentally obtained results are compiled in Table 1 and illustrated in Figure 1, Figure 2 and Figure 3 and related data enclosed in Supporting Materials.

The solvents’ effect in shaping micellar morphology in dispersion, as well as the dispersions’ concentration influence, both reflected on the films surface morphology are presented in Figure 1 and Figure 2 (S2–S4) and in Table S2. Thus, in Figure 1 are shown the AFM height images of the films’ topographic analysis obtained on glass, at a concentration of 1 mg/mL from both solvents. To get a better understanding of the shape and size of the nanostructures observed in this figure, it was considered useful to first analyze a larger surface area, to obtain a more realistic perspective on the surface morphology and how the visualized nano-objects were formed. So, the images of 60 × 60 μm^2^ scanned areas, for films from both solvents, are presented in Figure S3. At first glance, the appearance of film from Chl in Figure S3A(a) looks like a more uniform one by comparing with the one from DMSO in Figure S3B(a). It is covered with circular- and ovoidal-like particles, most of them of similar shape but of different size. The values of the apparent diameter or the length of the higher lateral dimension, of the biggest particles, are ranging from approximately 1 μm to approximately 9 μm (see the cross-sectional analysis in Figure S3B(a,b)).

The lower values of the thickness (between 100 and 400 nm) by comparing with their width, conduct to a relative high value of the aspect ratio (AR), (around 18/1); so, these nanoparticles are of a flat thick lamellar aspect and they most probably have been formed during the Chl evaporation and film forming by fusion of the smallest ones, which are still visible in the named figure.

As shown in Figure 1B(b,c), the measured values for the larger lateral dimension fall within the 100–500 nm range; additionally, particles in the process of fusing can be observed on the film surface (Figure 1A(a),B(a)).

It is important to highlight that these ovoidal nanoparticles, formed by casting from Chl, are of anisotropic character; they exhibit a thickness ranging from a few nanometers to several tens of nanometers (see the graphs for the given examples in Figure 1B(b,c)), with calculated AR values varying between 25 and 30. Consequently, their shape can be described as ovoidal lamellae.

It is generally accepted that as the solvent evaporates, the overall size of the supramolecular structure in the dry state decreases compared to the values recorded for the self-assembled structure while dispersed in a solvent. In Table 1, the values corresponding to the peaks in the intensity-weighted distribution traces (from Figure S2) are presented as particle sizes (nm).

It is important to highlight that DLS measurements obtained data must be considered with utmost care, in particular for those polymers for which the shape of the self-assembled structures most probably deviate from the spherical one, being anisotropic. More details on this aspect, besides the reason for our option, are given in Supplementary Materials.

If the results obtained from DLS measurements for OTh-PDLLA-1 are considered, (Table 1 and Figure S2A), which are in line with the previous reported ones related to CPs with similar architecture (see Supporting Materials for all mentioned details), they approximately correlate with the trends resulting from AFM investigations.

In the case of the film cast from DMSO, compared to the one obtained from Chl, the 60 μm^2^ surface area exhibits a morphology that appears significantly more heterogeneous and disordered, characterized by a mixed population of particles with varied shapes and diverse sizes (Figure S3A(b)). If one considers the particles with a more regular shape from Figure S3A(b)—which allows for a typical measurement—it can be observed that their dimensions are of the same order of magnitude as those obtained for Chl. Additionally, the cross-sectional analysis of the example shown in Figure S3B(c) indicates an AR of approximately 19/1, a value that falls within the same range as that of the Chl particles illustrated in Figure S3A(a).

Analyzing the morphology of the OTh-PDLLA-1 film cast from DMSO on smaller areas, of 10 × 10 μm^2^ and 5 × 5 μm^2^ (Figure 1C and Figure 1D), respectively, contributed to a better understanding on how this more polar and higher boiling point solvent influenced the co-oligomer’s self-assembling.

So, from Figure 1C(a) it can be seen that most of the formed nanostructures on the film surface have a top-view circular appearance, being placed in a highly organized manner, aligned like beads-on-string in a direction approximately parallel with the oy—axis; the 3D image of this scanned area, included in Figure S4(a), supports this assertion. As Figure S4(b) also shows, the measured nanoparticles present similar size of width (almost 300 nm), also having a constant thickness of only 25–30 nm (Figure S4(c)), resulting in a value of 10/1 for AR, which is characteristic to an anisotropic shape. Taken together, the value of AR, the shape of the graph in the cross-sectional analysis in Figure S4(c) and the thickness value less than 100 nm, all define these nanoparticles being of 2D type with a oblate dome shape.

In addition to these particles, which cover the area under investigation, other self-assembled structures—which are not spheroidal in shape—can also be discerned in the image shown in Figure 1C(a). The ones circled in pink, magnified and analyzed in Figure 1C(b,c), show an apparent ellipsoidal shape (see the 3D inset in Figure 1C(c)), with values of axial length, (Figure 1C(c)), and crosswise length of 1000 nm and 300 nm, respectively and a thickness of only 50 nm, resulting in a value of AR of 21/1.

All values for the aforementioned parameters indicate that these nanoparticles have an elongated, flattened, ellipsoidal shape. It can be assumed that such ellipsoidal micellar particles were already present in the OTh-PDLLA-1 dispersion in DMSO, possessing sufficient thermodynamic stability to retain their shape even after the solvent had evaporated.

The presence of particles with such anisotropy could be one of the reasons why no reliable results were obtained in the DLS measurement at this concentration (Table 1).

On the other hand, other self-assembled structures (such as those marked in blue in Figure 1C), which show edged faces and angles, most likely resulted by fusion via a hierarchical morphological evolution that occurred during solvent evaporation and film formation.

The fact that they retained a fixed shape, regardless of size, supports this claim. In Figure 1D(a) a decreased area is presented. In addition to the previously discussed one, in this figure much smaller circular structures (marked in green and denoted with number 1) with a discoidal shape have been also identified. The other described types (oblate dome-number 2, ellipsoidal-number 3), smaller in size are also found, populating the film surface. For a better understanding of the information included in Figure 1D(a), in Table S2, in Supporting Materials, all details about sizes and shapes are given.

Interestingly, in another area of the film scanned over a larger surface and illustrated in Figure S3A(b), a new supramolecular structure was identified, with a shape approaching a perfect hexagon; this is shown in Figure 1D.

Indeed, on a 5 × 5 μm^2^ square surface, taken as representative and presented in Figure 1D(b), it can be noticed that the film surface is covered with structures having shapes as that circled in green and magnified. They are of different size, with almost all of them having an integral hexagonal shape, arranged on the film surface in a “face-on” manner. Beside them, structures with non-integral hexagonal shape are present, too. The thickness of the exemplified hexagonal structure is almost 40 nm, while the value of the higher lateral dimension is 1.4 μm. The value of the ratio of axial length to crosswise length is 2/1 and the calculated value of AR resulted as 35/1 (from data in Figure 1D(b)). All of these values are in line with those typical for 2D nanostructures, which were denoted as “platelets”, whose shape is characterized by a distinctly edged or facetted contour [32]. Such 2D structures, in self-standing format, are mostly obtained from copolymers containing at least one crystallizable block, by applying crystallization-driven self-assembling (CDSA) in solution, in its various techniques [33]; this technique leverages the crystallization of the core-forming block to direct the formation of anisotropic 2D assemblies with low curvature.

Even if fewer in number, articles on the use of CPs as crystallizable components in combination with flexible counterparts in different topologies, forming 2D self-assembled platelets, have however been reported [34,35]. In order to understand the formation of the hexagonal platelets from Figure 1D(b), in the particular case of OTh-PDLLA, we considered previously reported results on surface-assisted self-assembly, such as 2D nano-objects generated by the interaction of oligothiophenes with substrates of various types [36], the occurrence of oligothiophene polymorphism typical for only film phase [37] and a more recent case of obtaining single crystals in film, with micrometric dimensions, from oligothiophenes solutions, under conditions comparable to those in which the OTh-PDLLA films were obtained [38].

Although the existence of 2D micelles in the dispersion in DMSO should not be completely ruled out, it is most likely that the hexagonal structures appeared during OTh-PDLLA-1 film formation, triggered by the character of DMSO, (as moderate solvent for the PDLLA side chains and non-solvent for the conjugated oligotiophene main chain), favored by the dynamic character of the process and by the procedure applied for films obtaining. Thus, the slow evaporation and high boiling point of DMSO can facilitate an extended period of interaction between the co-oligomer and the solvent, which would allow the rigid main chains to align and organize themselves more effectively. On the other hand, due to the dynamic increase in concentration during the evaporation of DMSO, enhanced co-oligomer—co-oligomer and co-oligomer—substrate interactions could promote the amplification of intermolecular π–π interactions between the main chains, acting as a driving force for self-assembly. In the evaporation scenario the main chains come closer due to the increasingly advanced collapse of the PDLLA side chains; collapse which, in addition, leads to a more efficient intra- and intermolecular interaction of the PDLLA’s terminal hydroxyl groups and to the eventual formation of hydrogen bonds between them, all together these processes would lead to the eventual crystallization of the oligothiophene rods and the stabilization of the entire supramolecular structure. It is noteworthy that these appeared as hexagonally-shaped structures on the OTh-PDLLA-1 film surface, and completed the “image” on how the polar DMSO influence the OTh-PDLLA self-assembling and films formation on glass support.

The influence of concentration was followed as well, and the morphology of the resulted films from both solvents, at a higher concentration of 10 mg/mL, is presented in Figure 2 and Figure S5. For the rod-*g*-coil copolymers self-assembly, a “rod–coil competitive mechanism” was proposed when molecular dynamics (MD) simulation was applied and, accordingly with the results, the cylindrical micelles are forming when the orientational order of the rod blocks predominates [39]. One from influence factors on this rod blocks’ orientational order is the concentration. These details are important for explaining the OTh-PDLLA’s films morphology at the ten times increasing concentration, in particular in the PDLLA’s good solvent Chl (Figure 2A). Thus, if looking at the AFM height image of the high length surface of 50 × 50 μm^2^ area in Figure 2A(a), seemingly there are no obvious differences regarding the size and shape of the self-assembled supramolecular structures when compared with those from Figure S3A of OTh-PDLLA-1 films. However, when focusing on the smaller area of 10 × 10 μm^2^ (Figure 2B(a) and Figure S5) well-organized, almost homogeneous in shape and uniformly sized interesting supramolecular structures can be noticed. They are toroids as the cross-sectional analysis in Figure 2B(b) proves.

The AFM height profile shows that the diameter of the exemplified toroid (D_toroid_) is approximately 200 nm, the diameter of the forming cylinder (d_toroid_) is around 100 nm, while the thickness is only 10 nm as a result of deflection during solvent evaporation. The interest in these types of nanoparticles is currently very high, as toroids belong to a morphology with promising applications in nanomedicine and materials science, in particular those derived from polymers self-assembling [32,40].

In terms of topological structure, toroids can be viewed as ring-shaped cylinders.

For rod-*g*-coil copolymers PBLG-*g*-PEG, it was found experimentally [41] and subsequently explained by computational studies [42] that derived semiflexible cylindrical micelles, having the rigid polymer backbones ordered parallel to the long axis of the micelle, can be bent into toroids via end-to-end coalescence. Based on the same end-to-end coalescence mechanism, more recently, toroids were reported formed from bottle brush copolymers possessing precisely tailored sequences and amphiphilicity [43].

Particularly important for this process is a feature these cylindrical micelles have, namely the presence of higher free-energy end-caps. They are resulting because the grafted side chains have no capability to totally cover them. A way to eliminate these unfavorable end-caps is the formation of toroids by simply bringing two ends of one cylinder together by what was denoted as “supramolecular cyclization” [42].

Regarding OTh-PDLLA, to understand why the toroidal structures formed (more clearly visible in Figure S5(a,b)), it is important to highlight that the molecular architecture and the rigidity of the main chain are features this polymacromonomer shares with the examples above. Also, bear in mind that the reported OTh-PDLLA co-oligomer belongs to the class of cylindrical polymer brushes, a name which highlights the peculiar supramolecular shape adopted by this type of architecture [44]. Furthermore, the ability of polythiophenes grafted with various side chains to form worm-like or rod-like micelles in solution has already been demonstrated [45,46]. To hypothesize that OTh-PDLLA also forms in Chl micelles with a rod-like appearance is a rational approach. In support of this claim comes the known oligomer nature of the conjugated main chain (see Experimental part) and OTh-PDLLA’s grafted architecture and, most importantly, the visual evidence from Figure 2A,B and Figure S5. Thus, it is well accepted that for semiflexible polymers (as CPs are [47]), when their contour length is smaller than their established persistence length, they act like rigid rods [48]. As the calculated length values of oligothiophenes (till septithiophene) [47,49] is less than the persistence length of, for example, poly(3-hexyl thiophene) [50], a rigid-rod-like behavior is expectable for oligothiophenes of such length.

The presence on the investigated film surface of the clearly discernible horseshoe-like structures (as that marked in green and magnified in Figure S5(a)) can be a visual evidence of the cylindrical micelles in Chl dispersion, as the spontaneous curvature of related cylindrical molecular brushes, when adsorbed on a flat surface, is a well-documented phenomenon in the literature [51]. A plausible scenario for the formation of OTh-PDLLA-10’s cylindrical micelles in Chl would be based on less extended PDLLA side chains by comparing with the case of the 1 mg/mL concentration due to a more crowded environment, which may favor more planar and rigid oligothiophene main chains, able to easily organize on a parallel direction with the long axis of a cylindrical micelle. Despite the visual evidence of toroidal structures on the surface of the OTh-PDLLA-10 film, the question remains whether the toroidal micelles actually exist in Chl solution or are formed during the casting and drying process. If we were to consider the structural peculiarities of OTh-PDLLA in the light of recently published results, based on computational simulation, regarding the self-assembly of amphiphilic comb-type copolymers, which claim the concomitant existence in solution of both cylindrical and toroidal micelles in the case of oligomers [52], we could assume that all discernible structures in Figure 2B(a) and Figure S5 were formed in solution. The result of the DLS analysis (Table 1 and Figure S2B) does not help us much in this regard, the value obtained for apparent hydrodynamic diameter (D_h_) being almost identical to that in the case of a concentration of 1 mg/mL. When referring to the literature examples, to the best of our knowledge, there are only a few works on the formation of toroidal structures by the conjugated polymers [53,54] and, as in the present case, their formation was determined by chance, without the molecular design of considered polymers having been intentional and guided by rules. In a more recent report [54], the nanotoroidal micelles were obtained by nanoprecipitation, being well-dispersed in water, while in a previous one, toroidal nanostructures derived from polyfluorenes were obtained by surface-assisted, evaporation-induced self-assembly, following an elaborate processing protocol [53]. Both are very different from our case. To get a well-documented conclusion on the formation of the noticed nanotoroids when OTh-PDLLA-10 was drop-cast from Chl on glass, a devoted study must be performed in the future in this regard. At the moment, considering the types of nanotoroids that populate the film surface given in Figure 2B(a), it could be stated that some of these structures could have formed by a morphological transition of cylindrical micelles in the dispersion state, by end-to-end coalescence, while other ones could have resulted on the support surface, from the cylindrical micelles, through their spontaneous bending and preferential fusion of the end-caps induced by solvent evaporation. Although not particularly high, the polydispersity index of the co-oligomer (PDI = 2) can significantly influence the self-assembly phenomenon [55], which explains the diversity in the sizes and shapes of the resulting supramolecular structures, as observed in Figure 2 and Figure S5.

It is also highly interesting that the film surface morphology obtained when OTh-PDLLA was cast from DMSO was at a concentration of 10 mg/mL. Figure 2C presents AFM height images captured at various scanning scales, which allow for the differentiation of various surface levels. The morphology’s general aspect in Figure 2C(a) is essentially different from those in Figure 1C,D obtained at a concentration of 1 mg/mL. At the higher concentration, the formed supramolecular structures have a similar anisotropic shape regardless of their size and, even though they are randomly scattered across the surface of the film, they are in the form of organized clusters. The images at lower length scale of 10 × 10 μm^2^ and 5 × 5 μm^2^ unveiled a homogeneous organization of the formed structures inside of those clusters. As can be seen from the AFM height image in Figure 2C(c), particles with an atypical shape, suggesting as being lamellar, all of them having the same size of their length around 500–530 nm (as it is marked with blue straight lines in the mentioned figure) are present, all of them being tilted in the same direction. The measured thickness of one from these particles (enclosed within the green rectangle, marked with the number 1 in Figure 2C(c) and magnified) resulted around 40 nm (see the graph of the cross-section below the magnified image). The calculated AR of 13/1 supports their classification as lamellae. The magnified image also reveals that these lamellar structures associate in a partially overlapping manner and, as can indeed be noticed in both images 2C(b,c), they are acting as fundamental building blocks. These AFM results suggest that, when dispersed in DMSO at 10 mg/mL, a hierarchical organization of OTh-PDLLA’s self-assembled structures occurs from solution to thin film. In fact, the DLS measurement results given in Table 1 for this sample would suggest an anisotropic shape of both the particles and the clusters formation (Figure S2C). During solvent evaporation and film formation, a surface-mediated self-assembly process and morphological evolution may have occurred, resulting in structures formed by the fusion of elementary units (see the micron size structure highlighted in the green square labeled with “2” in Figure 2C(c)). With an increased length and thickness, these supramolecular structures resulted with a similar AR value as the cross-sectional analysis proved.

For films cast on glass from both solvents, the concentration exerts a significant effect on the contact angle, with an increase in hydrophobicity observed as the concentration rises from 1 to 10 mg/mL. In Table 1, in addition to the values of root mean square roughness S_q_, the values of two other parameters characteristic to 3D surface topography, as Wenzel roughness factor **r** and developed interfacial surface area ratio S_dr_ (based on AFM data) are listed as well, as they turned out to be the indicators of choice for correlating with the wettability of a polymer surface [56]. From the data in Table 1 it can be noticed that there are similar trends of the above-named parameters with a concentration variation in WCA values.

To determine the influence of the type of the support on the film surface morphology, OTh-PDLLA was cast onto PLA sheets, from DMSO, at both specified concentrations, following the procedure described in detail in the experimental section.

By analyzing the data in Table 1 regarding the wettability of films cast on PLA support, the variation in WCA values is slight—only 3 degrees—and the value decreases as the concentration increases. Interestingly, a similar variation was also determined for factor **r** and for S_dr_ values, a trend in stark contrast with the evolution when glass slides were used for films casting. This fact highlights the importance of the used support on the self-assembled film morphology, determining the wetting behavior.

The results of the AFM investigation of films cast from DMSO on PLA sheets are given in Figure 3. At the lowest concentration of 1 mg/mL the image in Figure 3A(a) and the graph of cross-sectional analysis in Figure 3A(b) reveal a layered morphology. A very important aspect is that these results demonstrate that PLA support maintained its integrity during the film processing, confirming the claim already stated in the introduction.

Additionally, a parallel experiment was conducted by applying a volume of DMSO identical to the amount used in the preparation of the co-oligomer films onto a bare PLA sheet; this sheet was subsequently processed in the same manner as for the OTh-PDLLA films (see the Experimental section). In Figure S6 are shown the AFM images of surfaces of bare PLA sheet, of DMSO-treated PLA sheet and those of the OTh-PDLLA-1 and OTh-PDLLA-10 films of a larger scanned area of 50–60 μm^2^. If the images labeled **a** and **c** in Figure S6A are compared, except several thin crack lines discernible on the DMSO-treated PLA sheet surface (which possibly appeared during the vacuum drying and are due to a slight swelling of the upper PLA surface layer in DMSO), the general appearance is similar to that of untreated PLA. This conclusion is enhanced by the morphology at lower scale of 10 × 10 μm^2^ (Figure S6A(b,d)) for which the size and shape of the structures that appeared are very similar. In Figure S6B(a,b), the AFM height images are presenting the heterogeneous and concentration-dependent morphology of the OTh-PDLLA films at a larger scale, which emphasize both the clear identity and integrity of the PLA support and the deposited films.

The images from Figure 3A(c,d) add new information to all of the above. Thus, on the surface layer, platelets-like structures of micrometric size are visible, most of them in regular triangular shape (marked with blue lines in Figure 3A(c)), arranged in a “face-on” manner in relation to support and placed one on top of the other, as can be seen in Figure 3A(c,d).

The thickness of the measured one (see Figure 3A(d) cross-sectional graph) is approximately of 25 nm, which resulted in a calculated value of AR of 36/1, characterizing 2D platelets. Such triangular structures with their shape with sharp edges and angles allude to those usually formed by PLA stereocomplexes in the case of an imbalanced PLA system [57]. Given that the PLA support consists of 96% PLLA, one might speculate that a specific interaction, occurring during solution casting, involving segments of the PDLLA oligomeric side chains—rich in one of the enantiomers—and solvent-swelled PLLA chains from the support’s upper layer could have led to the formation of these types of lamellar structures, in a manner as recently reported [58]. On the surface of the superficial layer pits were also detected, which most probably formed during the DMSO slow evaporation. The measured dimensions given in Figure 3A(c) of such a hole are almost 25 nm in depth and a diameter of high value of 600 nm. By comparing all of these results with those obtained with OTh-PDLLA-1 on a glass slide, significant differences can be noticed in all aspects. Thus, regarding the surface morphology of the films, hexagonally-shaped 2D platelets—which are slightly thicker—appear only sporadically on the glass substrate. More pronounced differences are observed regarding the wettability and roughness parameter values (**r** and S_dr_ in Table 1); in the case of the PLA support, these parameters did not vary monotonically with respect to the concentration values.

When the concentration of OTh-PDLLA in DMO increased at 10 mg/mL, as expected, the morphology of thin film surface changed. However, as with glass, the structures formed on PLA exhibit the same shape regardless of size (Figure 3B(b,c)). The resulting self-assembled structures present on the film surface are lamellar in this case, as well. A tendency toward clusterization was also noticed as can be seen more evident in Figure 3B(a). As in the case of glass, some of the formed lamellae can be considered as building blocks for the larger formed structures. They have almost identical length of 500 nm as can be seen in Figure 3B(b) and in the magnified images derived from the regions in Figure 3B(c) marked with green rectangles. These so-called “building blocks lamellae” are remarkably thinner, the measured thickness being only 3–4 nm as the cross-sectional analysis shows in magnified image. Most of the formed lamellar structures are arranged in the “face-on “manner, have a planar surface (see cross-sectional graph in Figure 3B(b)) and, as in the case of glass these lamellar structures associate in a partially overlapping manner (Figure 3B(b,c)). While WCA value only decreased slightly (3 degrees) with the concentration increase, the decreasing in value of topographical parameters (r and S_dr_) was more significant with the concentration increase (Table 1), having an inverse trend as that noticed for glass support.

### 3.2. Study of the OTh-PDLLA S Thin Film Morphology and Surface Properties Changes upon the Addition of LiClO_4_ by Solution Mixing

By considering native ionic signaling of biological systems communication, a new class of CPs-based materials has been developed able to simultaneously transport and couple ionic and electronic charge carriers, commonly referred to as organic/polymeric mixed ionic-electronic conductors (OMIEC/PMIEC) to be used in biointerfacing [59]. The main design criteria, which have to be considered for OMIEC optimally performing in an aqueous physiological environment, are: (i)—to “make ions flow” [60] and (ii)—to allow for electrochemically aqueous electrolytes compatibility [25]. Different polar side chains, appended to electron conducting hydrophobic π-conjugated backbone, that promote ions solvation/coordination, forming a percolating electrolyte phase that mediates doping and ionic conduction, were reported [61]. However, despite their critical role, design rules for mixed conducting CPs side chains are still nascent and their effects on PMIEC morphology have yet to be systematically investigated [62].

In this context, it was assumed that OTh-PDLLA surface properties can be modulated by changing the polar PDLLA side chains’ hydrophilicity [62], with direct impact on its performance as biomaterial. The inorganic lithium perchlorate (LiClO_4_) was chosen to modify the grafted co-oligomer in its chloroform solution. This option was based on our own, but also of others, previous results, related to polythiophene or PEDOT copolymers considered for bioapplications, that were electrochemically synthesized using LiClO_4_ as the supporting electrolyte, and for which it has been convincingly demonstrated that, while serving as dopant source, it does not negatively impact the biocompatibility of the obtained materials [63,64,65,66,67]. In addition, we took into account the successful attempts of utilizing PLA salted with LiClO_4_ (bare or as blend of polymers) as “non-traditional” solid polymer ionic electrolytes [68,69,70]. This was taken as a benefit of OTh-PDLLA architecture, the grafted PDLLA side chains getting by this transformation as an ionic character due to Li^+^ cations coordination, which could function as a communication channel between the solid film and ionic biological media, promoting the flow of ions and water molecules at the biotic/abiotic interface. In other words, it was considered that this modification could transform OTh-PDLLA into an efficient OMIEC material.

**Figure 4:**
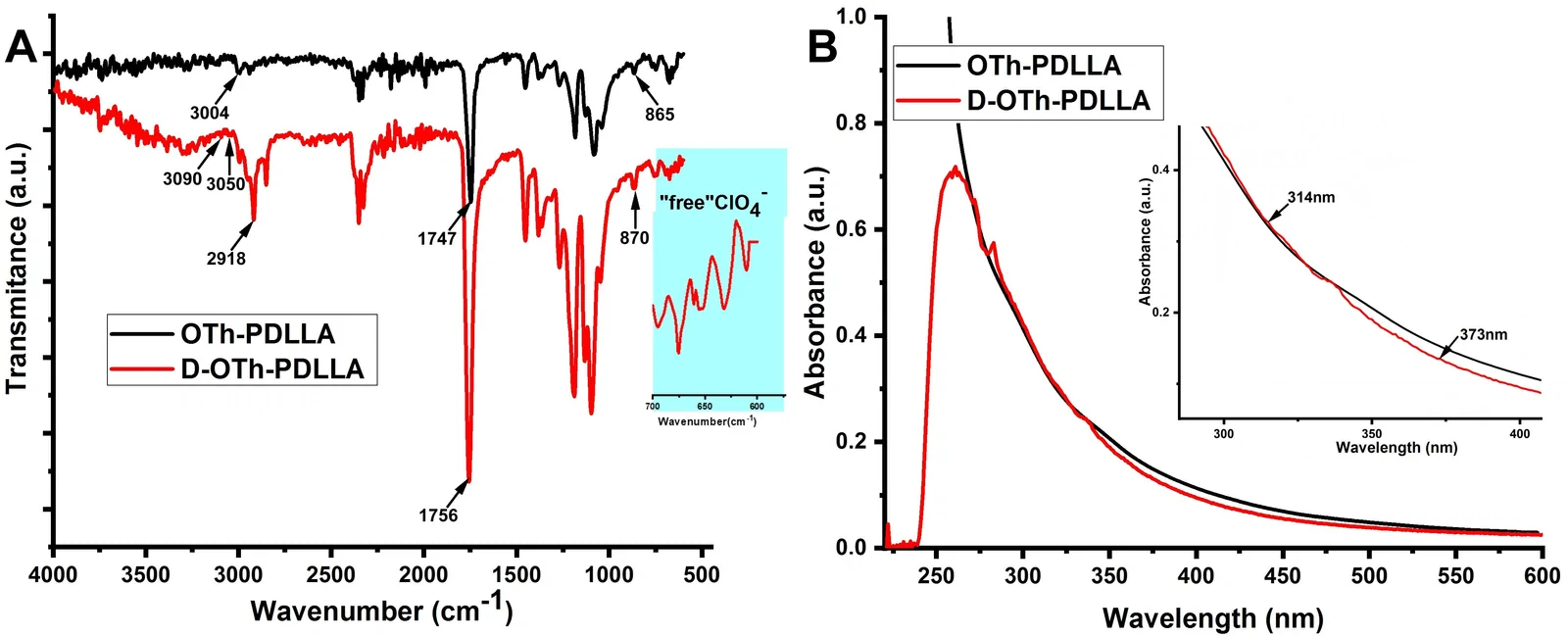
(**A**)—ATR-FTIR spectra of OTh-PDLLA and D-OTh-DLLA films drop cast on glass support from Chl, at concentration of 1 mg/mL; (**B**)—UV-vis absorption spectra of OTh-PDLLA and D-OTh-PDLLA registered in Chl at concentration of 1 mg/mL.

The successful coordination/intercalation of LiClO_4_ into the OTh-PDLLA matrix was confirmed both in film by ATR-FTIR and in solution, by UV-vis absorption spectrophotometry (Figure 4). As shown in the ATR-FTIR spectrum of the resulting product denoted D-OTh-PDLLA (Figure 4A-red line), besides the characteristic absorption bands associated with the structure of native OTh-PDLLA [24], one of the most obvious differences is related to the stretching vibration mode of the carbonyl group (υ(C=O)) in ester functionalities of PDLLA, which shifts from 1747 cm^−1^ to a higher wavenumber at 1756 cm^−1^. This change in the stretching frequency position could be most probable due to the coordination between the carbonyl group of the side chains oligolactide backbone and lithium cations [70]. The Li^+^ coordination changes the conformations of the PDLLA side chains. This claim is supported by the noticed modification in the ATR-FTIR spectrum of D-OTh-PDLLA film compared with that of the native oligomer OTh-PDLLA in the range 2800–3100 cm^−1^. This region is characteristic to the appearance of the valence stretching vibrations of both the aliphatic-CH_3_ (ν_as_(CH_3_) and ν_s_(CH_3_)) and of -CH moieties, respectively from the skeleton of the PDLLA side chains. The two traces’ shape and bands intensity significantly vary for OTh-PDLLA and D-OTh-PDLLA with the signal’s positions being shifted to the lower wavenumbers for the LiClO_4_—treated sample.

Notably, it is the position and the intensity of the band at 2918 cm^−1^, attributed to ν_s_(CH_3_) in D-OTh-PDLLA in comparison with the homologous one in OTh-PDLLA with a lower intensity and placed at 2940 cm^−1^. Also, in the same region, the bands specific to aromatic C_α_-H and C_β_-H stretching vibration of the thienyl rings are discernible for both films but in different shapes and positions, namely at 3090 cm^−1^ and 3050 cm^−1^ in D-OTh-PDLLA spectrum, while in that of OTh-PDLLA a broad band centered at 3004 cm^−1^ could be assigned to both the thienyl rings in the backbone and to ν_as_(CH_3_) stretching vibrations due to the side chains. These last described absorptions, typical for aromatics, prove that the main chain’s environment was changed during the LiClO_4_—treatment. In support of this assumption also comes the presence of peaks typical for conjugated oligothiophenic backbone [24], that appeared slightly shifted in the LiClO_4_—modified film (at 870 cm^−1^) by comparing with the native film at 865 cm^−1^.

Not less important is the presence in the spectrum of D-OTh-PDLLA of new small signals emerged in the range 674–611 cm^−1^ (marked and enlarged in the inset of Figure 4A), which were previously assigned to the vibrations of “free” perchlorate anions, υ(ClO_4_^−^) [71]. These small splitted bands could be correlated with the presence of a broad and shallow one in the range 3200–3600 cm^−1^ attributable to the hydroxyl end groups of PDLLA side chains. Such a band is not visible in the spectrum of OTh-PDLLA, inducing the idea that the surface of the native film deposited in the similar conditions misses the presence of these PDLLA side chains’ hydroxyl end-groups. These experimental findings correlate with the results of contact angles determinations shown in Table 1, confirming the enhanced hydrophilicity of the D-OTh-PDLLA film surface.

The effect induced by the presence of LiClO_4_ coordinated to D-OTh-PDLLA was also investigated by the changes registered on its electronic state as resulted from the UV-vis measurements (Figure 4B). As can be seen, prior the modification, the UV-vis absorptions of the conjugated moieties of OTh-PDLLA were scarcely discernible due to a phenomenon also noticed previously and explained by the unique architecture of OTh-PDLLA containing high grafting density of the side-chains [24,45].

In contrast, in the UV-vis spectrum of D-OTh-PDLLA, a well-defined, broad and asymmetric trace was obtained showing a maximum at 260 nm and some discernible shoulders at 283, 320 and 337 nm. In addition, in the range 314–375 nm (Figure 4B-inset) some other shallow bands appeared as the magnified inset, as Figure 4B shows.

**Figure 6:**
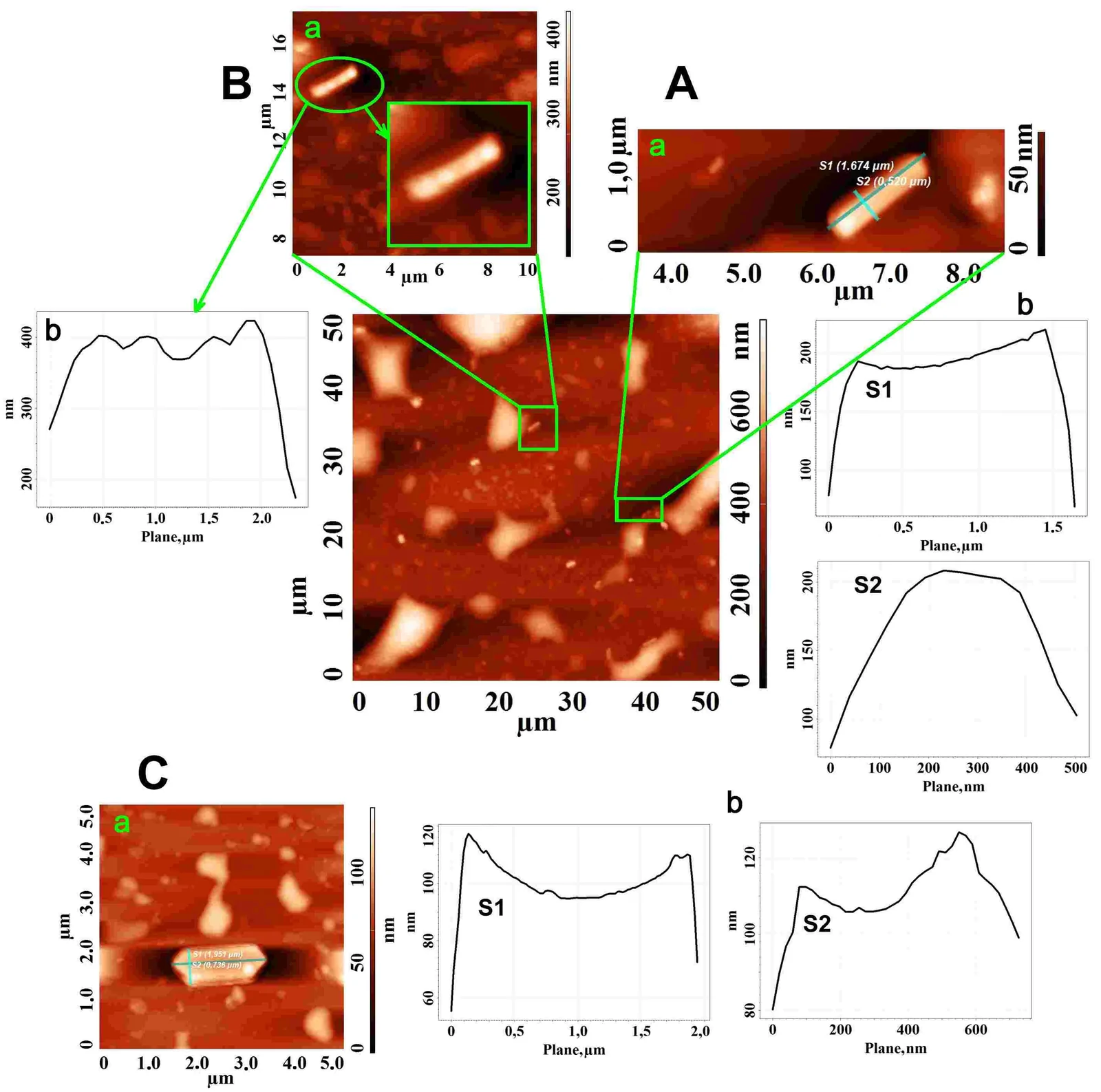
AFM height images and cross-sectional analysis of film surface morphology obtained by drop-casing of D-OTh-PDLLA on glass, dispersed in Chl, at concentration of 1 mg/mL, showing platelets the size of which fall in a range from microns to tens of microns and most of them being of random shape (central image), other ones of well-defined rectangular platelets (**A**) formed by fusion of spherical-like particles (**B**) and hexagonally-shaped platelets stacked one over the other (**C**), all of them with a value of thickness ranging between 50 nm and 250 nm.

The modification of the photophysical properties of aromatic chromophores-containing compounds, in organic solvents, induced by the Li^+^ cations have been reported in the literature. These alterations were induced, for example, by the coordination of Li^+^ by the ether-containing moieties appended to the aromatic chromophores in the structure of the investigated compounds [72]. In the particular case of D-OTh-PDLLA, the change in UV-vis behavior could be due to the conformational change in the oligolactide side chains when hosting Li^+^ cations through coordination; most likely, they became much more extended and a conformational change in the conjugated oligothiophene main chain is also possible. As a result of these modifications the size and shape of the self-assembled particles formed in Chl by D-OTh-PDLLA are different from those formed by the co-oligomer in its native state. This claim is being supported by the AFM images from Figure 1A and Figure 6 that show very different types of morphologies for OTh-PDLLA and D-OTh-PDLLA films, respectively, drop-cast from Chl in similar conditions. These images are the proof that size- and shape-different micellar nanoparticles are forming in Chl in native and in LiClO_4_—treated state, which could behave differently in the UV-vis measurement. The noticed maximum in the UV-vis trace of D-OTh-PDLLA is most probably given by the carbonyl moiety from the PDLLA side chains, as previously determined [24], while the other mentioned shoulders, in particular those from the higher wavelengths region could be attributed to the oligothiophene backbone, probably much more twisted in D-OTh-PDLLA due to enhanced repulsive interactions of the highly dense, and more extended Li^+^-coordinated side chains.

**Figure 5:**
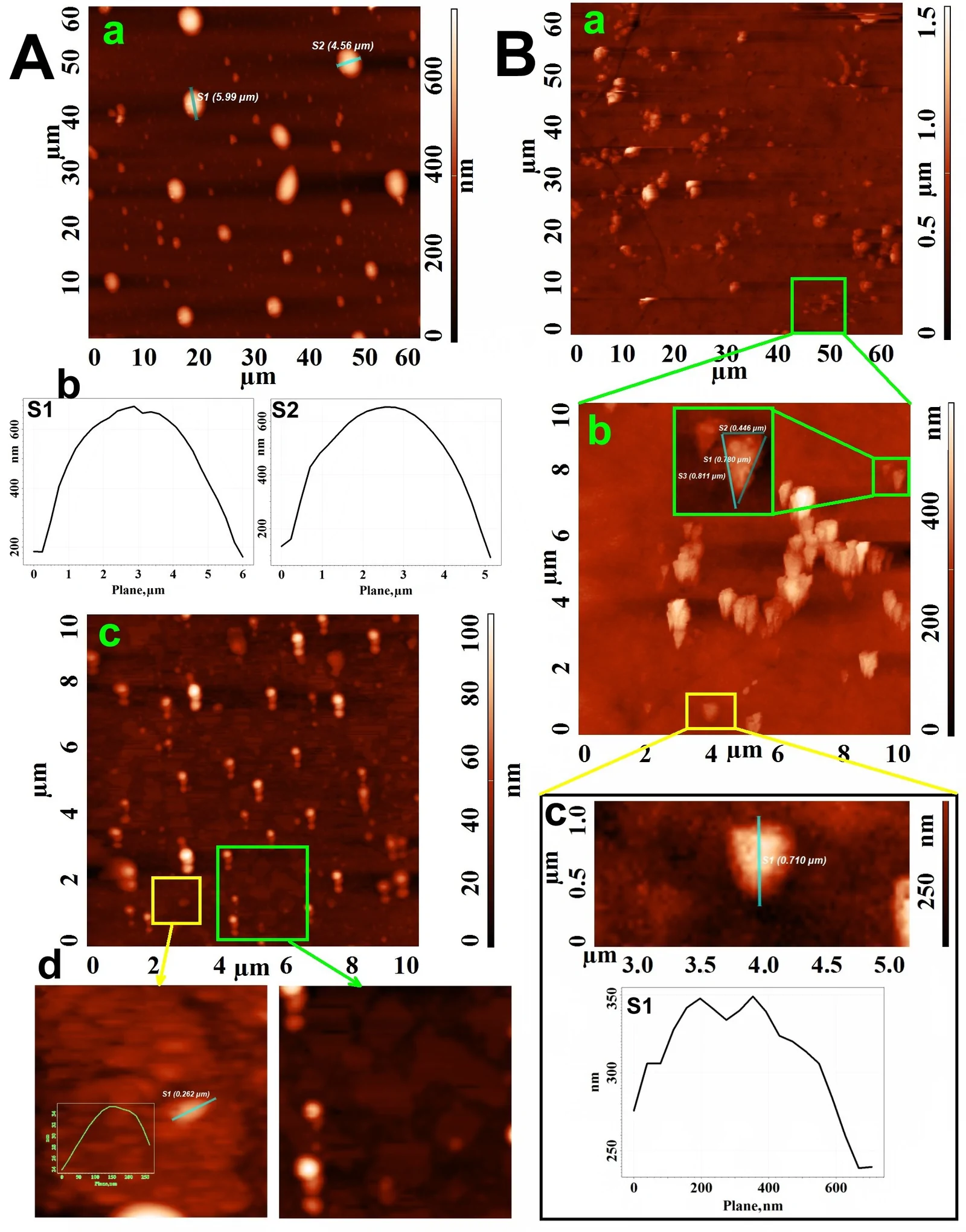
(**A**)—AFM height image of film surface morphology formed onto glass slide by the drop-casting of D-OTh-PDLLA dispersion in DMSO at concentration of 1 mg/mL (**A**(**a**,**c**,**d**)) and cross-sectional analysis of the largest formed ovoidal lamellae; (**A**(**b**)); (**B**)—AFM height image of film surface morphology drop-cast on PLA sheet from D-OTh-PDLLA dispersion in DMSO at concentration of 1 mg/mL (**A**(**a**)) with detailed triangular shape and size analysis (**B**(**b**,**c**)).

The changes of film surface properties induced by the LiClO_4_ in bulk treatment by mixing solutions were further investigated by water contact angle measurement and visually by AFM (Table 1 and Figure 5 and Figure 6), considering the films obtained by drop casting from DMSO on glass and PLA sheets and from Chl on glass, all of them obtained using the concentration of 1 mg/mL.

As can be seen from the data in Table 1, by comparing with the films derived from native OTh-PDLLA obtained in similar conditions, the wettability of those derived from D-OTh-PDLLA increased as the WCA values decreased. Thus, after Li^+^-coordination by the PDLLA side chains the WCA were reduced with 18–29° by comparing with the native films, highlighting the versatile ability of the synthesized oligomer to enhance the surface hydrophilic character through a simple and easy processing modification. This increased hydrophilic character is influenced (as the data from Table 1 show) by the solvent nature and by the type of the support. It is more pronounced for glass support when cast from more polar DMSO. The effect of the Li^+^-coordination by the PDLLA side chains on the topographical parameters **r** and S_dr_ is also solvent- and support-dependent. Thus, the increase in hydrophilicity is accompanied by an increase in the roughness parameters for Chl on glass and for DMSO on PLA, while a decrease in the roughness can be noticed for film of D-OTh-PDLLA casted from DMSO on glass. In addition, it was found that by using the same solvent DMSO and varying only the casting supports both wettability and films roughness can be significantly modulated, the smoother film of D-OTh-PDLLA on glass (r = 1.005 ± 0.001) being more hydrophilic by comparing with that formed on PLA sheet, which turned out to be much rougher (**r** = 1.08107 ± 0.015). These findings induced the idea that the presence of the ions on the film surfaces is the decisive influence factor for the increased hydrophilicity of D-OTh-PDLLA by comparing with the native similar films, while other surface characteristics as surface chemical composition or surface crystallinity are accompanying the surface polarity and surface charge.

AFM investigations shed light on the self-assembling features for D-OTh-PDLLA films and the impact of LiClO_4_ treatment on the films surface morphology. Thus, in Figure 5 are presented, in a mirror manner, the film surface characteristics of D-OTh-PDLLA obtained on glass (Figure 5A) and on PLA sheet (Figure 5B).

The first observation is that the presence of LiClO_4_ coordinated to oligomer structure induced, as expected, the morphological changes of the film surfaces when compared with those of native OTh-PDLLA drop-cast on the similar supports at the same concentration (Figure 1C,D and Figure 3A). However, the most obvious are the differences induced by the support nature on the D-OTh-PDLLA’s film morphologies, which contribute to their different hydrophilicity. Thus, as can be seen in Figure 5A(a), on the film surface deposited on glass, ovoidal-shaped particles of different sizes are present, randomly distributed on the entire investigated area, the biggest ones having the length of the microns order and a thickness of approximately 400 nm (Figure 5A(b)). By decreasing the scrutinized area at 10 × 10 μm^2^, new details appeared as Figure 5A(c) shows.

Interestingly, a layered morphology is discernible, with a superficial layer formed by groups having three globular particles, each one of different size, but which together form a similar, well-defined pattern, regardless of their size (Figure 5A(c)). Moreover, these groups are aligned on trajectories, which are oriented toward the left side in oblique directions relative to the film surface. The presence and abundance of such organized structures induce the idea that they were formed during the DMSO slow evaporation, by fusion of smaller basic particles, resulting in one from the most thermodynamically stable arrangements.

The basal layer formed around these three particles-forming groups have an appearance of island-like areas of ill-defined geometric shapes, with edges and angles as can be seen in the green marked area in Figure 5A(c), which is magnified in Figure 5A(d) indicated with the green arrow. The intimate morphology of such basal layers is formed from small ovoidal lamellae arranged in a regular “fish scales” manner, as exemplified in the area marked in yellow in Figure 5A(c) and magnified in Figure 5A(d) indicated with the yellow arrow.

Measuring one of the most prominent from these lamellae resulted in a length of approximately 250 nm and a thickness of 10 nm as the cross-sectional graph in green in Figure 5A(d) shows. This arrangement and general appearance of rounded particles on surfaces of all film layers could be the motivation for the lowest value of the roughness factor **r** of this film compared to all other ones investigated in this study.

By contrast, at the first sight, the surface of the film deposited on the PLA sheet is characterized by clusters of particles, randomly distributed, many of which having an edgy appearance (Figure 5B(a)). In a smallest area presented in Figure 5B(b) are clearly discernable triangular platelets, which are singularly spread across the entire surface of the film or such platelets are assembled forming clusters of various sizes, in which they are arranged one over the other, suggesting a hierarchical organization. When comparing with the triangular platelets formed on the film surface of native OTh-PDLLA on the PLA sheet (Figure 3A, highlighted with straight blue lines), the ones from the green and yellow marked area in Figure 5B(b) and detailed in Figure 5B(c) have almost similar dimensions (around 500–900 nm) with those from Figure 3A, but they show different aspect ratio and different manner of the arrangement onto the surface of the film. These different peculiarities of the films surface morphology could lie at the base for the differences noticed in the roughness parameters between treated and native films on PLA sheets and between films of D-OTh-PDLLA drop-cast on glass and on PLA sheets, respectively (see Table 1).

As previously mentioned in this paper, the formation of these well-defined triangular structures could be promoted by the combination of favoring factors, such as the high boiling point of DMSO determining a slow evaporation and the PLA sheets derived from 96% L-enantiomer containing 2003D PLA.

Changing the solvent from DMSO to Chl, the film obtained from D-OTh-PDLLA on glass (central image in Figure 6) have a totally different morphology by comparing with its homologue obtained from DMSO (Figure 5A(a)).

It is characterized by the presence on the film surface of platelets of tens of microns as size (the biggest ones) with sharp edges and angles but with irregular geometrical forms. Besides them, smaller particles with rectangular rod-like appearances are visible, as well (green marked areas in the central image of Figure 6). Focusing on these structures (Figure 6A(a)) it was revealed that they are rectangular platelets with values of length varying between 1.5 and 2 μm, width around 500–600 nm and thickness of 100–250 nm (Figure 6A(b),B(b)). They were formed by fusion of globular nanoparticles as it is revealed by the green-marked magnified excerpt in Figure 6B(a).

Not only regularly shaped rectangular platelets were discernible on this film surface, but also hexagonal ones as that presented in Figure 6C(a), having the values of length and width in the same range but thinner than the rectangular platelets. Taking into account the morphologies of the film surfaces obtained from Chl at different concentrations and cast on various supports investigated through this paper, it can be concluded that salt LiClO_4_ was the main promoter that drives the formation of these well-organized and defined structures.

## 4. Conclusions

All in all, the above results unveiled a versatile capability of the presented oligomer OTh-PDLLA to be processed as functional surface coatings with modifiable and adaptable properties driven by the self-assembling. The strong rigidity contrast between the rod and coil segments of the grafted co-oligomer can diminish the influence of their volume fractions such that thermodynamic factors dominate in the self-assembly of the formed nanostructures. It is also very important to consider the CPs’ crystallization mechanism, in segmented copolymers, induced by the self-assembling and in addition the solution-state out-of-equilibrium polymer assemblies, which are controlled by kinetic factors.

The main findings of the present study are as follows:

- All the studied influencing factors, which are important in film processing, affect the size and shape of the self-assembled structures and the film surface properties of OTh-PDLLA co-oligomer;
- By varying the concentration of both considered solvents, an increase in WCA and in the roughness parameters values with the concentration increase was noticed in the case of glass as support; an inverse trend for the named film surface characteristics was noticed when OTh-PDLLA was cast from DMSO on PLA sheets;
- The solvent type changing, despite greatly affecting the self-assembled nanostructures on the film surface, which were reflected in the values of the roughness parameters, only slightly influenced the values of WCA for both OTh-PDLLA-1 and OTh-PDLLA-10;
- When DMSO was used to obtain OTh-PDLLA-10 films, a similar pattern of self-assembly behavior was observed, regardless of the substrate employed. Specifically, lamellar supramolecular structures formed on both substrates; these structures exhibited well-defined shapes that remained identical in form, irrespective of their size. The surface morphology of the resulting films revealed that these structures evolved toward a stable morphology through a process of hierarchical association;
- On glass slides, switching the solvent from Chl to DMSO results in a transition from a film surface containing a mixed population of particles, varying in shape and size, to a more organized appearance of surface morphology, which was reflected in a significant increase in the roughness value but a very low variation in WCA;
- By treating OTh-PDLLA with LiClO_4_, in bulk by mixing solutions, regardless of the solvent and the used support, the film surface of resulted D-OTh-PDLLA presented an enhanced hydrophilicity with the values of WCA varying between 49 and 65°;
- DMSO as a moderate solvent for PDLLA side chains, LiClO_4_ as a modifying salt and PLA sheets as support, all favored the formation, by surface-assisted self- assembly, of 2D lamellar structures, some of them of a very regular shape (hexagonal, rectangular, triangular).

## Figures and Tables

**Table 1:** The values of WCA and of roughness parameters (Sq, r, Sdr) by AFM analysis of studied films and peaks value of the intensity-weighted particles’ size distribution obtained for dispersions of co-oligomer in Chl or DMSO by DLS measurements.

Sample	Average Water Contact Angle (Degree) Mean		^2^ Average Roughness by AFM Sq (nm)		^3^ Mean Roughness Factor r and (Sdr %)		Average Particles Size (nm)
	Glass ^1^ (78)	PLA Sheet ^1^ (82)	Glass (2.55)	PLA Sheet (36.5)	Glass	PLA Sheet	
OTh-PDLLA-1 Chl	74 ± 3.7	-	7 ± 0.4	-	1.00234 ± 0.0001 (0.234 ± 0.01)		937 ± 93.75 (100%)
OTh-PDLLA-10 Chl	85 ± 1.2	-	15 ± 1.4	-	1.00348 ± 0.0011 (0.348 ± 0.11)		947 ± 13.42 (100%)
D-OTh-PDLLA Chl	56 ± 2.1	-	18 ± 1.7	-	1.00757 ± 0.0017 (0.757 ± 0.17)		n.d.
OTh-PDLLA-1 DMSO	78 ± 2.6	83 ± 1.6	19 ± 3.08	14 ± 2.3	1.01668 ± 0.005 (1.668 ± 0.5)	1.01616 ± 0.0045 (1.616 ± 0.45)	*
OTh-PDLLA-10 DMSO	83 ± 1.4	80 ± 2.4	53 ± 4.9	73 ± 5.9	1.01907 ± 0.005 (1.907 ± 0.5)	1.00475 ± 0.001 (0.475 ± 0.1)	602 ± 72.24 (64%); 5100 ± 20.59 (36%)
D-OTh-PDLLA- DMSO	49 ± 3.6	65 ± 9.1	9 ± 0.7	33 ± 5.1	1.00544 ± 0.0014 (0.544 ± 0.14)	1.08107 ± 0.015 (8.107 ± 1.5)	n.d.

^1^ Values of WCA of the uncovered supports; ^2^ Sq—root mean square roughness for a 10 × 10 µm^2^ scanned area; ^3^ r—calculated as the ratio between the geometric (S3A) and projected surface (S2A). Values are reported as mean ± standard deviation (n = 3 different regions scans of single film); Sdr—developed surface area as given by Image Analysis 1.0.0.0. based on the formula: (S3A − S2A)/S2A; Values are reported as mean ± standard deviation (n = 3 different regions scans of single film); n.d.—not determinated; *—not reliable results were obtained from the measurements.

## Data Availability

The raw data supporting the conclusions of this article will be made available by the authors on request.

## Supplementary Materials

The following supporting information can be downloaded at: https://www.mdpi.com/article/10.3390/polym18212724/s1, Figure S1. Digital image of OTh-PDLLA-10 film drop-cast on glass from Chl; Table S1. Several physical properties of the used solvents for OTh-PDLLA self-assembling and casting and of PDLLA side chains; Figure S2. DLS traces of intensity-weighted distribution of apparent hydrodynamic diameter (D_h_) for OTh-PDLLA in (A) Chl at concentration of 1 mg/mL; (B) Chl at concentration of 10 mg/mL; and (C) DMSO at concentration of 10 mg/mL; in the inset of each image are enclosed the values of Z-average size and standard deviations obtained for self-assembled particles; DLS comments; Figure S3. AFM height images (A) and the traces of cross-sectional analysis (B) of the nanostructures formed on the film surfaces of OTh-PDLLA deposited from Chl and DMSO, at concentration of 1 mg/mL, scrutinized on a surface area of 60 × 60 μm^2^; Figure S4. AFM of OTh-PDLLA-1 cast from DMSO on glass: (a)—3D image (a) of 10 × 10 μm^2^ investigated area; (b)—AFM height image with marked section for beads-of-string-like nanoparticles; (c)—Cross-sectional trace of the marked particles in figure (b); Figure S5. (a)—AFM height image of OTh-PDLLA-10 film surface cast on glass from Chl, showing uniformly sized toroids; (b)—AFM 3D image of a magnified area showing an individual toroid; Table S2. Details on the size and shape of the nanostructures presented in Figure 1D(a); Figure S6. (A-a and b)—AFM height image of bare PLA sheet used for OTh-PDLLA films deposition; (A-c and d)—AFM height image of bare PLA sheet of similar size as those used for film deposition, treated with 30 μL DMSO and handled in a similar manner as for films obtainment (see Experimental Part); (B)—(a)—AFM height image of OTh-PDLLA film cast on PLA sheet from DMSO at concentration of 1 mg/mL and (b) from DMSO at a concentration of 10 mg/mL; For further details one may consult the references [20,21,48,49,50,73,74,75,76,77,78,79,80].
