# Supplementary material for: Oligothiophene-g-oligo(D,L-lactide) “Rod-Graft-Coil” Co-Oligomer: Self-Assembly Modulation by Processing and Formed Films Surface Morphological Diversity Characterization
Source: Polymers (Basel). 2026 Nov 2;18(21):2724. doi: 10.3390/polym18212724 (PMC13765631; doi:10.3390/polym18212724)
Supplement: Supplementary file 1 [file polymers-18-02724-s001.zip › polymers-4630812-supplementary.pdf]

Supplementary Materials

Oligothiophene-*g*-oligo(D,L-lactide) "Rod-*graft*-Coil" co-oligomer: Self-assembly modulation by processing and formed films surface morphological diversity characterization

Anca-Dana Bendrea[a,§], Demet Göen-Colak[b, §], Luminita Cianga[a,*], Mariana Pinteala[a], Ioan Cianga[a,*]

[a] Centre of Advanced Research in Bionanoconjugates and Biopolymers,"Petru Poni" Institute of Macromolecular Chemistry 41 A, Grigore-GhicaVoda Alley, 700487 Iasi, Romania; anca.bendrea@icmpp.ro; lcianga@icmpp.ro; pinteala @icmpp.ro; ioanc@icmpp.ro

[b] Istanbul Technical University, Faculty of Science and Letters, Department of Chemistry, Maslak, 34469 Istanbul, Turkey; goende@itu.edu.tr

§- equally contributed to this work

[*] Corresponding authors at "Petru Poni" Institute of Macromolecular Chemistry, Iasi, Romania

E-mail addresses: lcianga@icmpp.ro (L. Cianga); ioanc@icmpp.ro (I. Cianga)

EXPERIMENTAL SECTION-Supporting figure

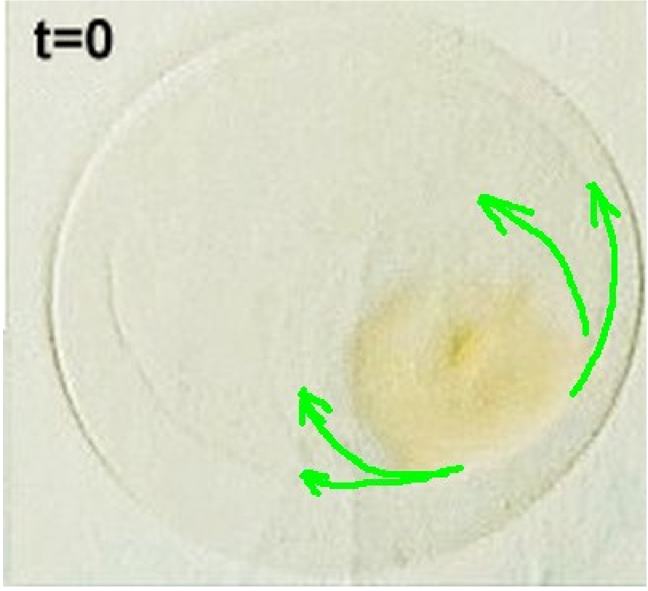

Figure S1. Digital image of OTh-PDLLA-10 film drop-casted on glass from Chl

RESULTS AND DISCUSSION-Supporting figures, tables, comments and references

Table S1. Several physical properties of the used solvents for OTh-PDLLA self-assembling and casting and of PDLLA side chains*

**Compound**	**Hansen solubility parameters ($MPa^{1/2}$) and molar volume V(cm^3x mol^{-1})**					**Flory-Huggins polymer-solvent interaction parameter (χ_{p-s})#**	
	**δ_D**	**δ_P**	**δ_H**	**δ_t**	**V**	**Chl**	**DMSO**
**PDLLA**	18.6[1]	9.9[1]	6[1]	22[2]/20.5[3]	-	0.398	0.432
**Chl**	17.8[4]	3.1[4]	5.7[4]	19[4]	80.7[4]		
**DMSO**	18.4[4]	16.4[4]	10.2[4]	26.7[4]	71.3[4]		

*-The superscipt numbers in the table correspond to the similar numbers in References section;#- χ_{p-s} was calculated by using the formula reported in reference **5**, considering the value of correction parameter α as being equal with 1 and the room temperature T (°C) at which the experiments were performed as 25°C.

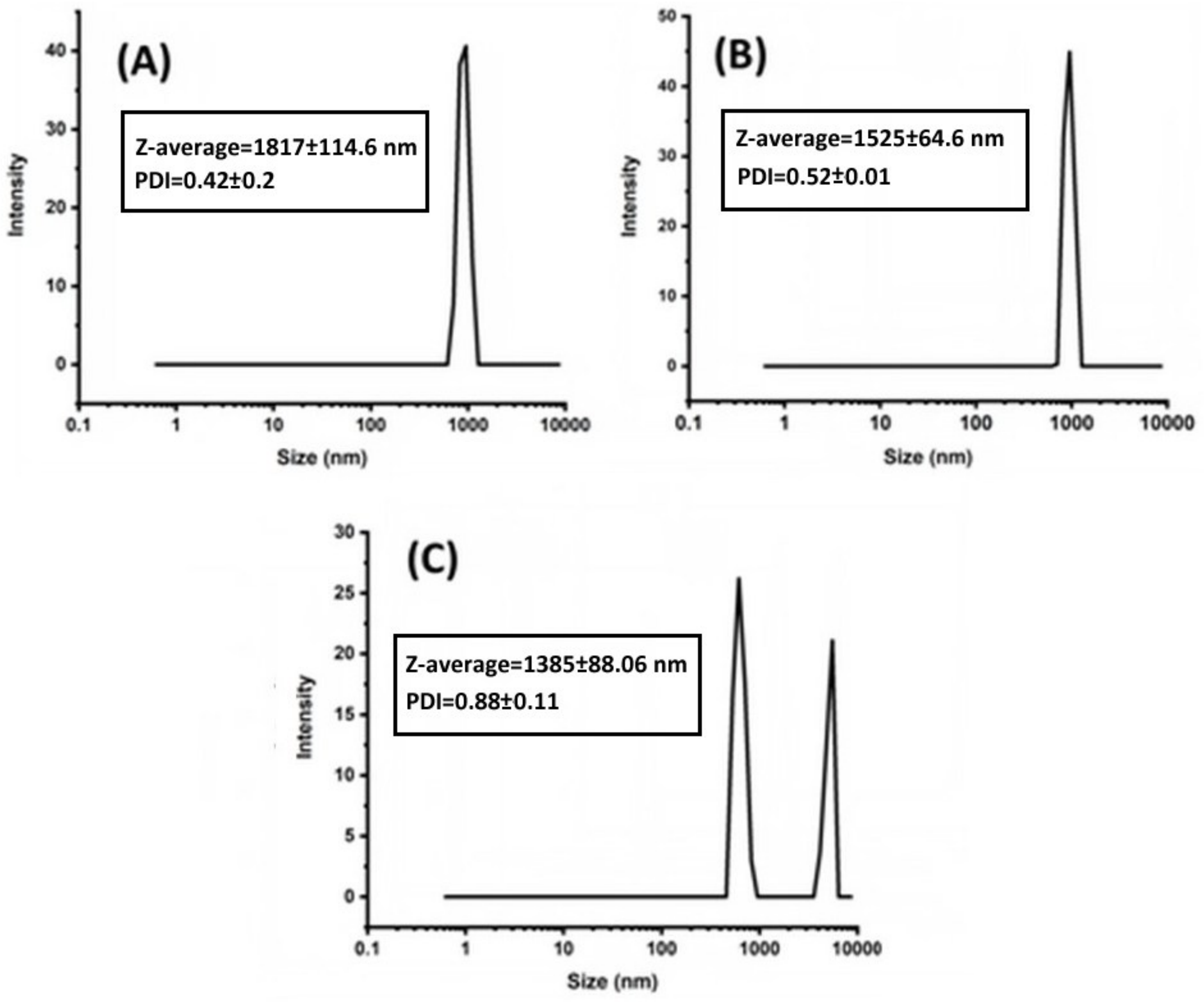

Figure S2. DLS traces of intensity-weighted distribution of apparent hydrodynamic diameter (D_h) for **OTh-PDLLA** in (**A**) Chl at concentration of 1mg/mL; (**B**) Chl at concentration of 10 mg/mL; and (**C**) DMSO at concentration of 10mg/mL; in the inset of each image are enclosed the values of Z-average size and standard deviations obtained for self-assembled particles

DLS comments

The values obtained from DLS measurements must be considered with care as this technique is based on the assumptions that the particles are non-interacting spheres and not anisotropic objects, and that the particle size is the diameter of the sphere diffusing at the same speed as the particles being measured [6]. Due to the complex architecture OTh-PDLLA have, the prediction of the shape of the self-assembled micellar structure in different organic solvents is difficult for many reasons. These are including, beside solvophilicity of the co-oligomer structural building blocks, the rigidity and shape-contrast amphiphilicity and conjugated main chain's capacity for crystallization. However, based on the known oligomeric short length of the rigid main chain [7], for which the value of the contour length can be significantly lower

than that of the persistence length of, for example poly-3 hexyl thiophene (l_p = 3 nm [8]), that is classified as a semiflexible polymer, it is not out of rational to predict that OTh-PDLLA would behave in dispersion as the rigid rods [9], conducting to formation of micelles of various anisotropic shape which subsequently, after drying and film forming, could evolve in various types of self-assembled structures; one of them having more regular shape (as for example horse-shoe-like, toroid-like, ovoidal or platelets shape) another ones of irregular forms but with visual evidences that they derived by a hierarchical surface-mediated morphological transition, as AFM investigation revealed. In this case, the high polydispersity of the size given by the DLS measurement-quality information makes the Z-average values of particles size not suitable for a reliable data interpretation (see the reported values for each case in the insets in Figure S2). For this reason we have aligned our approach with previous reports [10-13] - specifically those involving formation by self-assembling of non-spherical anisotropic supramolecular structures [12, 13] - and have considered the values of the maxima of the intensity-weighted distribution traces as the particle size (Table 1- main manuscript).

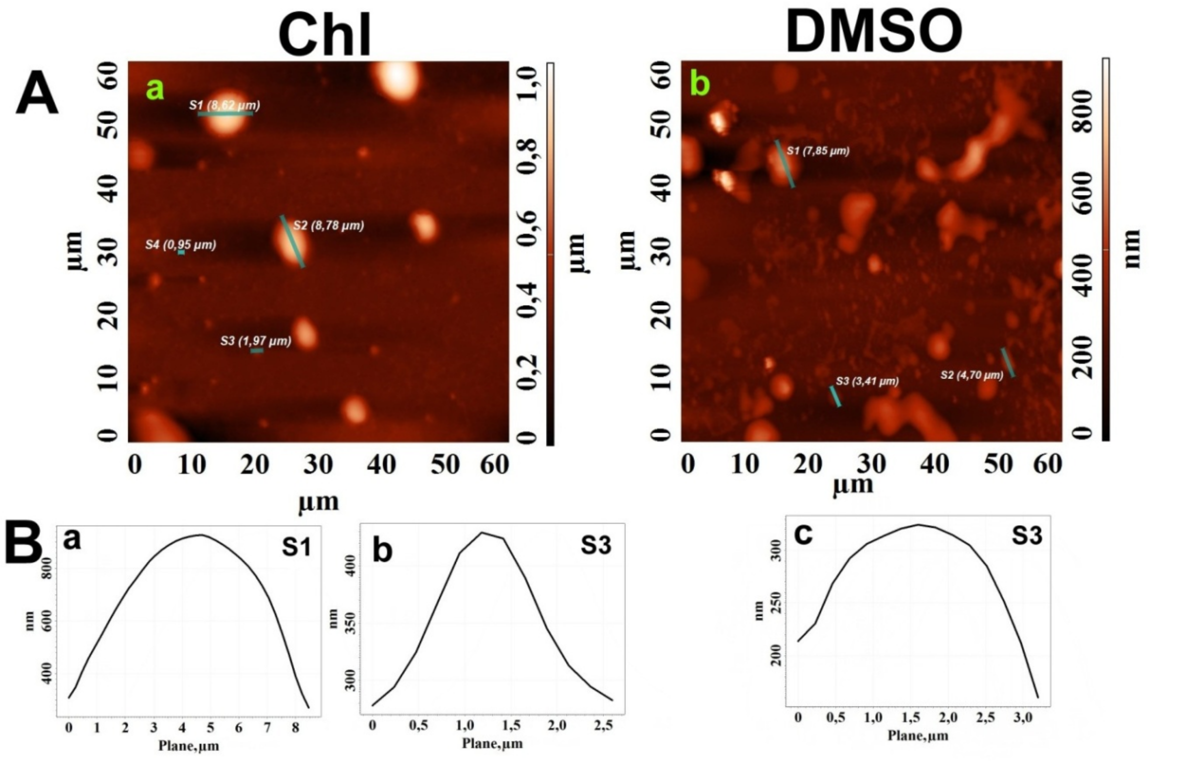

Figure S3. AFM height images (**A**) and the traces of cross-sectional analysis (**B**) of the nanostructures formed on the film surfaces of OTh-PDLLA deposited from Chl and DMSO, at concentration of 1 mg/mL, scrutinized on a surface area of 60x60 μm^2

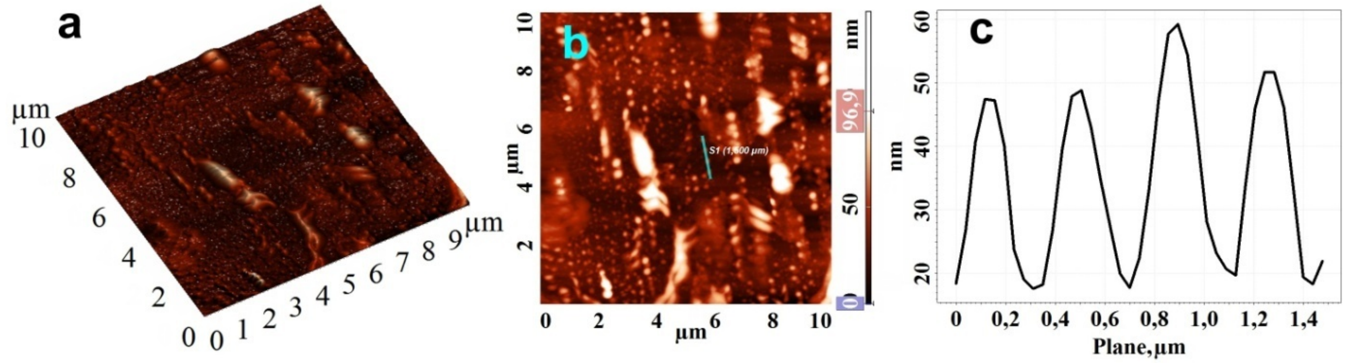

Figure S4. AFM of OTh-PDLLA-1 casted from DMSO on glass: (a)- 3D image (a) of 10x10 μm^2 investigated area; (b)- AFM height image with marked section for beads-of-string-like nanoparticles; (c)- Cross-sectional trace of the marked particles in figure (b)

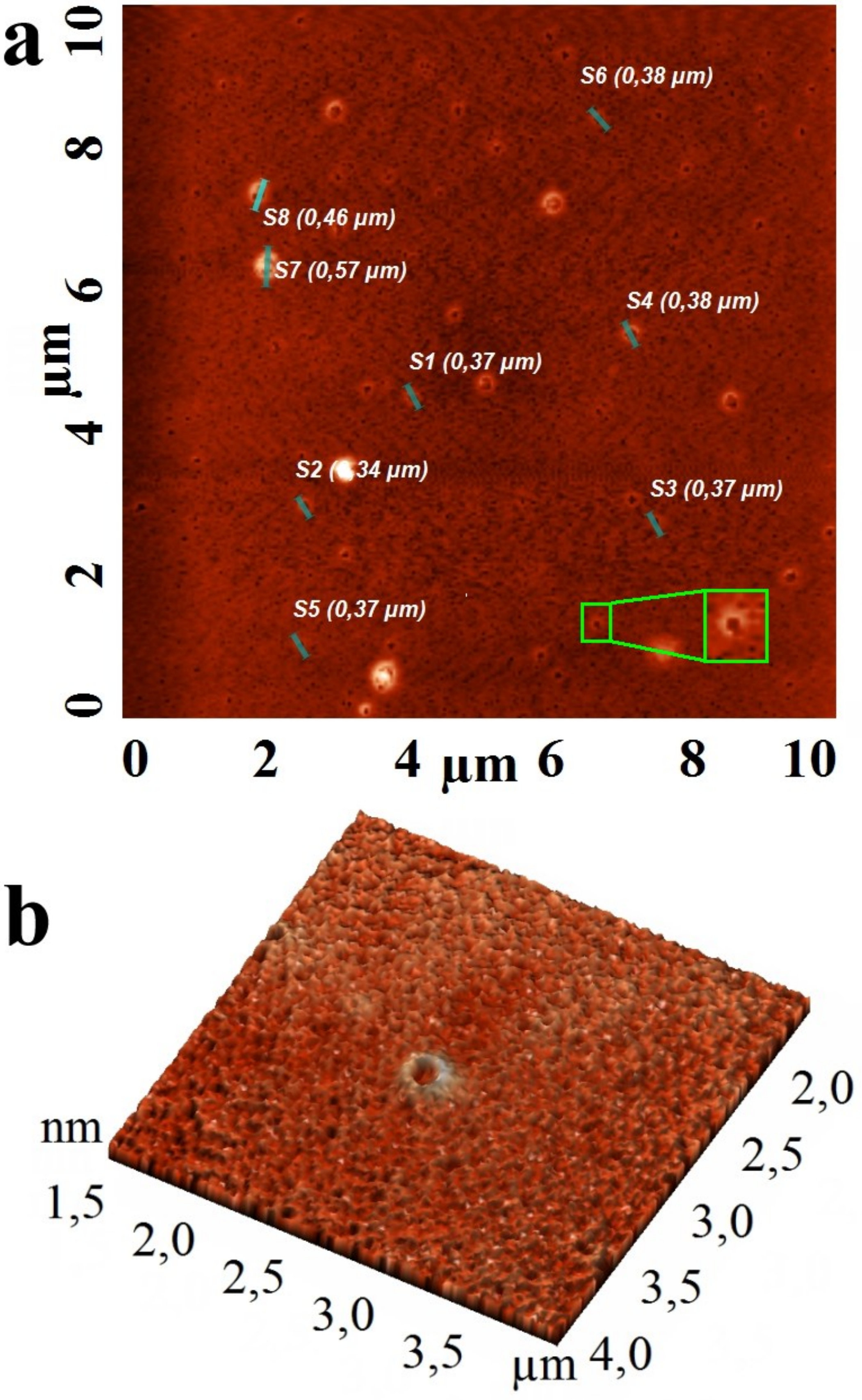

Figure **S5**. (**a**)- AFM height image of OTh-PDLLA-10 film surface casted on glass from Chl, showing uniformly sized toroids; (b)- AFM 3D image of a magnified area showing an individual toroid

Table S2. Details on the size and shape of the nanostructures presented in **Figure 1-D (a)**

**Particle**	**Length (nm)**	**Thickness (nm)**	**Aspect ratio (length/thickness)**	**Shape**
**1**	79	4	20/1	Discoidal
**2**	274	30	9/1	Oblate dome
**3**	633	18	35/1	Ellipsoidal/prolate

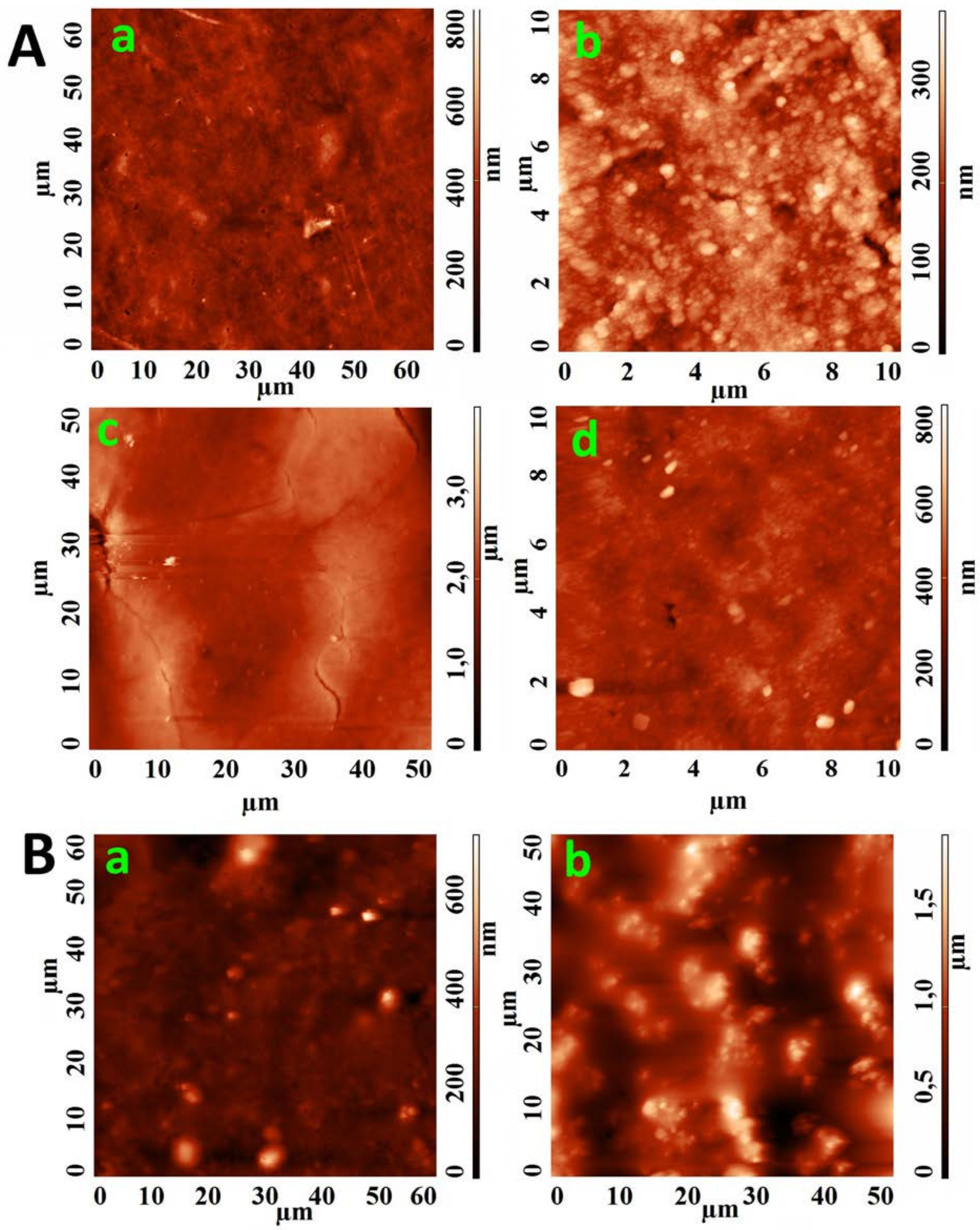

Figure S6. (**A-a** and **b**)- AFM height image of bare PLA sheet used for OTh-PDLLA films deposition; (**A-c** and **d**)- AFM height image of bare PLA sheet of similar size as those used for film deposition, treated with 30 μL DMSO and handled in a similar manner as for films obtainment (see Experimental Part); (**B**)- (**a**)- AFM height image of OTh-PDLLA film casted on PLA sheet from DMSO at concentration of 1 mg/mL and (**b**) from DMSO at a concentration of 10 mg/mL

References

1. S. Abbott, Chemical compatibility of poly(lactic acid): A practical framework using Hansen solubility parameters, in Poly(lactic acid) Synthesis, Structure, Properties, Processing, and Application, R. Auras, et al., Editors. 2010, John Wiley and Sons, Inc.: Hoboken, New Jersey. p. 83-89.

2. K. Adamska, A. Voelkel, A. Berlinska, The solubility parameter for biomedical polymers-Application of inverse gas chromatography, *J. Pharm. Biomed*., 2016, 127, 202-206.

3. U. Siemann, The solubility parameter of poly(DL-lactic acid), Eur. Polym. J., 1992, 28, 293-297.

4. A. F. Barton, Solubility Parameters, *Chem. Rev*., 1975, 75, 731-753.

5. 6. Lindvig, T.; Michelsen, M. L.; Kontogeorgis, G. M. A Flory−Huggins Model Based on the Hansen Solubility Parameters. *Fluid Phase Equilib.* 2002, 203, 247−260.

6. D. G. Colak, I. Cianga, L. Cianga, Y. Yagci. Synthesis and self-assembly of fluorene-vinylene alternating copolymers in “Hairy-Rod” architecture: side chain – mediated tuning of conformation, microstructure and photophysical properties. Des. Monom. Polym., 2016, 19(6), 508-534.

7. R. Lin; M. Galili; U. J. Quaade; M. Brandbyge; T. Bjornholm; A. D. Esposti; F. Biscarini; K. Stokbro, Spontaneous Dissociation of a Conjugated Molecule on the Si(100) Surface. *J. Chem. Phys.* 2002, *117*, 321-330.

8. K. Gu, J. Onorato, S. S. Xiao, C. K. Luscombe, and Y.-L. Loo, Determination of the Molecular Weight of Conjugated Polymers with Diffusion-Ordered NMR Spectroscopy, Chem. Mater. 2018, 30, 570-576.

9. L. Gerrits,; R. Hammink,; P. H. Kouwer, Semiflexible Polymer Scaffolds: An Overview of Conjugation Strategies. *Polym. Chem.* 2021, 12, 1362-1392.

10. U. Halder, S. Mondal, S. Hazra, S. Guin, L. Yeasmin, Tailor made synthesis of water-soluble polythiophene-graft-poly(caprolactone-block-dimethylaminoethyl methacrylate) copolymer and their pH tunable self-assembly and optoelectronic properties, Eur. Polym. J. 168 (2022) 11112, https://doi.org/10.1016/j.eurpolymj.2022.111124.

11. F. Liu F, D. Wang, M. Zhang, L. Ma , C-Y. Yu, H. Wei, Synthesis of enzyme-responsive theranostic amphiphilic conjugated bottlebrush copolymers for enhanced anticancer drug delivery. Acta Biomater 2022;144:15–31. https://doi.org/10.1016/j.actbio.2022.03.028.

12. Rizis, G.; van de Ven, T. G.; Eisenberg, A. Raft" Formation by Two-Dimensional Self-Assembly of Block Copolymer Rod Micelles in Aqueous Solution. Angew. Chem., Int. Ed. 2014, 53, 9000-9003.
13. Wu, D.; Xu, F.; Huang, Y.; Chen, C.; Yu, C.; Feng, X.; Yan, D.; Mai, Y. Effect of Side Chains on the Low-Dimensional Self-Assembly of Polyphenylene-Based "Rod-Coil" Graft Copolymers in Solution. *Macromolecules* 2018, 51, 161-172.
